# Comparative Molecular and Epidemiological Analyses of Israeli Bluetongue Viruses Serotype 1 and 9 Causing Outbreaks in 2018–2020

**DOI:** 10.3390/microorganisms11020366

**Published:** 2023-02-01

**Authors:** Natalia Golender, Eyal Klement, Anita Kovtunenko, Boris Even-Tov, Lior Zamir, Eitan Tiomkin, Gabriel Kenigswald, Bernd Hoffmann

**Affiliations:** 1 Department of Virology, Kimron Veterinary Institute, Bet Dagan 5025000, Israel; 2 Koret School of Veterinary Medicine, The Robert H. Smith Faculty of Agriculture, Food & Environment, The Hebrew University of Jerusalem, P.O. Box 12, Rehovot 7610001, Israel; 3 Veterinary Servises in the Field, Galil-Golan 1231400, Israel; 4 Hachaklait Veterinary Services, Caesarea 3088900, Israel; 5 Institute of Diagnostic Virology, Friedrich-Loeffler-Institut, 17493 Greifswald-Insel Riems, Germany

**Keywords:** *Reoviridae*, *Orbivirus*, bluetongue virus, cattle, sheep, ruminants, descriptive epidemiology, sequencing, phylogeny

## Abstract

Israel is endemic to bluetongue virus (BTV). The introduction of novel-for-the-region arboviruses have been recorded annually in recent years. In 2019, previously non-reported in-the-country BTV-1 and BTV-9 were identified. BTV-1 caused a single-season outbreak, probably linked to mild infection in ruminants. BTV-9 was retrospectively detected in the field samples collected from August 2018 until 2020. It was the dominant serotype in 2019, out of the six serotypes recorded during that calendar year. Clinical manifestation of the disease in cases diagnosed with BTV-9 were compared to those in cases determined to have BTV-1. BLAST and phylogenetic analyses of BTV-1 showed that the nucleotide (nt) sequence coding the viral outer protein 1 (VP2) determining the serotype is closely related to BTV-1 isolated in Sudan in 1987, and the coding sequence of the outer protein 2 (VP5) is related to South African BTV-1 from 2017. A probable common ancestor with Libyan BTV-9 strains isolated in 2008 was seen in an analysis of Israeli BTV-9 nt sequences. Notably, the outbreak-caused BTV-9 strains collected in 2019 exhibited a distinct level of genetic reassortment with local Israeli strains compared to BTV-9 strains registered in 2018 and 2020.

## 1. Introduction

Bluetongue (BT) is a non-contagious, arthropod-borne infectious viral disease of domesticated and wild ruminants caused by bluetongue virus (BTV) and having worldwide distribution. This disease is most commonly observed in sheep and white-tailed deer, causing severe clinical manifestations and death, especially in naïve animals [1]. Transmission of most BTV serotypes between mammalian hosts relies on competent blood-feeding midges of the *Culicoides* species [1]. Vertical and horizontal transmissions have been described or/and hypothesized, but are considered to be of less epidemiological importance [2,3,4,5,6,7]. Severe clinical manifestations of the disease with frequent acute deaths are primarily seen in sheep and some wildlife species, while the infection is generally sub-clinical in cattle and goats. BT in susceptible animals is characterized by fever, hemorrhages, milk reduction, stiff gate, nasal congestion and discharges, oral erosions, coronitis, and facial/neck edema. BT-associated lesions include the following: hemorrhages and ulcers in the oral cavity and upper gastrointestinal tract; necrosis of skeletal and cardiac muscles; coronitis, subintimal hemorrhages in the pulmonary artery; oedema of the lungs, ventral subcutis, and fascia of the muscles of the neck and abdominal wall; and pericardial, pleural, and abdominal effusions [8].

BTV is a member of the *Orbivirus* genus within the *Reoviridae* family. The genome of BTV is composed of 10 linear double-stranded segments (Seg-1 to Seg-10) encoding seven structural (VP1 to VP7) and five nonstructural (NS1 to NS5) proteins [9]. Based on Seg-2 gene sequences and virus neutralization tests, currently 36 distinct BTV serotypes have been officially recognized [10,11].

BTV-1 has been frequently identified in many regions of the world. It is endemic serotype in some African countries (South Africa, North African countries) [12,13] and India [14,15]. In Australia, BTV-1 was the most frequent isolated serotype over thirty years (1979–2011) [16]. It was also identified and isolated in Eastern Asia (China and South Korea) [17,18] and even in South America (French Guiana) [19]. Since 2001, BTV-1 caused multiple outbreaks in several countries in Europe [20,21,22]. Moreover, it is still circulating in the European region. During 2020–2021 the serotypes 1, 2, 4, 8, 9, and 16 were identified in Europe, whereby BTV-1 was circulating in Italy and Corsica (France) only [23]. Regarding countries neighboring Israel, serological data from collected samples in 2011 showed presence of BTV-1 in Lebanon [24].

In a similar manner to BTV-1, BTV-9 was also identified at all continents. Thus, the first South African BTV-9 isolates are dated to 1942 (GenBank). Later BTV-9 was described by Gerdes et al. [12]. Regarding North African countries, BTV-9 was isolated in Libya in 2008. Notably, data on serological investigations showed its recent presence in this country [25]. In Europe, BTV-9 were registered for the first time in October 1998, on four Greek islands close to the Anatolian coast of Turkey. Afterwards BTV-9 were recorded between June and October 1999 as the cause of outbreaks in southeast Bulgaria [26]. According to the data submitted to the GenBank, BTV-9 was found in Kosovo (2001) and Italy (2003). In Asia, BTV-9 was recently serologically identified in India [27], when several BTV-9 submitted to the GenBank genome sequences were dated 2003–2007, which indicated their earlier presence in the country. Since the 1980s, BTV-9 has been identified in Australia [28]. BTV-9 was also detected in both American continents [29,30]. Regarding the Middle East region, BTV-9 was identified serologically in Syria and Jordan in 1978–1981 and in Turkey in 1980–1981. Additionally, in Turkey it caused outbreaks during 1999–2001 [31,32].

Israel is situated in very special geographic location among three continents. Close proximity to Africa, Asia, and Europe provides an opportunity to arboviral infections to spread to regions from all these directions. During the last decade many arboviruses belonging to different viral families, which previously had not been found here, were detected and isolated. Since 2014 a big number of different simbuviruses, having veterinary importance mostly as pathogens involved in pregnancy abnormalities among small and big domestic ruminants [33,34,35] and other non-BTV orbiviruses, such as several different epizootic hemorrhagic disease virus (EHDV) serotypes [36,37], have been reported. Almost all of them illustrated a close relationship with African strains except Akabane viruses (AKAV), which illustrated a strong similarity to Turkish AKAV [35], answering the question as to their origin and probable route of introduction.

Similarly, most recently detected new-for-Israel BTV strains probably have an African origin. To date, thirteen BTV serotypes have been registered in field samples collected from wild and domestic ruminant population in Israel: BTV-1, -2, -3, -4, -5, -6, -8, -9, -10, -12, -15, -16, and -24 [38,39,40]. During the period of the study (2018–2020), nine different serotypes—BTV-1, -2, -3, -4, -6, -8, -9, -12 and -15—were detected or/and isolated. Considering appearance of novel-for-Israel strains and serotypes, we can specifically sign the BTV-3 as becoming endemic Israeli serotype. However, we cannot be sure whether BTV-3 has been circulating in Israel since 2013 and has been registered annually since 2016, or whether closely related BTV-3 strains were frequently introduced into the region during the last years [35,39]. In 2017, a novel BTV-6 strain caused an outbreak in Israeli livestock when an insufficient number of successful BTV-6 virus isolation took place in 2018 and 2020 [35]. During the same time, “Nigerian” BTV-8 was identified in 2019 in two sheep only, which surprisingly did not spread through the country [39]. In 2020, a novel BTV-12 strain, being the dominant serotype during the summer of 2021, showed the closest relationship to the BTV-12 from Zambia (collection date 2018, GenBank). Regarding BTV-1 and BTV-9, both had never been identified before 2018 in the country. The aims of this study are the following: (i) outbreak description and clinical manifestation of the disease in susceptible Israeli ruminant population caused by BTV-1 and -9 registered between 2018–2020; (ii) comparison of their spread among Israeli ruminants; (iii) phylogenetic analyses of identified BTV-1 and -9.

## 2. Materials and Methods

### 2.1. Field Samples

A total of 3371 samples from 3111 animals (ill and dead domestic and wild/zoo ruminant, and aborted or malformed domestic or wild ruminant fetuses) submitted between 2018 and 2020 for routine examination to the virology department of the Kimron Veterinary Institute, Israel (KVI), were included in this study. Clinical specimens comprised placenta, brain, and internal organs from aborted fetuses, whole blood from symptomatic, and spleen or lung from dead ruminants. Data on field samples tested for BTV in 2018–2020 are summarized in Table 1.

### 2.2. Virus Isolation (VI)

The majority of BTV RT-qPCR positive samples collected between 2018 and 2020 and found suitable for virus isolation were inoculated into embryonated chicken eggs (ECE) according to the method described by Komarov and Goldsmit [41] (total number of 564 used samples). Isolated in ECE, part of BTVs were subsequently adapted to Vero (African green monkey kidney epithelial cells) or BHK-21 (baby hamster kidney cells).

### 2.3. Nucleic Acid Extraction and Pan-BTV Real-Time Polymerase Chain Reaction (RT-PCR)

We extracted ribonucleic acid (RNA) from the tissue culture supernatant, chicken embryo homogenates, and field samples (whole blood, lung, brain, spleen) with Invisorb Spin Virus RNA Mini Kit (STRATEC Molecular GmbH, Berlin, Germany), and MagMAX™ CORE Nucleic Acid Purification Kit (Thermo Fisher Scientific Austin, Texas, USA). Viral RNA detection was performed with VetMAX™ BTV NS3 All Genotypes Kit (Applied Biosystems™, Thermo Fisher Scientific Inc., Lissieu, France). Alternatively, the pan-BTV system described by Wernike et al. [42], which is also based on detection of Seg-10 fragment, has been employed. In accordance with the instructions of the authors and manufacturer of the RT-qPCR kit/system, the cutoff for all these methods was the Cycle Threshold (Ct) 40.

### 2.4. Type-Specific RT-PCRs

During 2018–2020, the samples identified as positive for BTV, with RT-qPCR and with Ct values up to 34, were further tested for determining the serotype. All pan-BTV positive samples collected during calendar years 2019–2020 were tested retrospectively for BTV-1, while positive samples from calendar years 2018–2020 were retrospectively tested in BTV-1 and BTV-9 specific RT-qPCRs. The presence of BTV-3 was tested by an in-house specific RT-qPCR according to the method described by Lorusso et al. [43], when tests for BTV-1, 4, -8, -9 and -15 were performed according to the method described by Maan et al. [44]. BTV isolates were typed by in-house conventional RT-PCR and by previously described method [39] and confirmed by sequencing of the PCR product. The One-Step RT-PCR kit (Qiagen, Hilden, Germany) was used for all conventional RT-PCRs.

### 2.5. Sequencing and Phylogenetic Analyses

Primers used for partial sequencing of BTV-1 and -9 are listed in Supplementary Table S1. The cDNA fragments of positive samples were purified with the MEGAquick-spin Total Fragment DNA Purification Kit (iNtRON Biotechnology, Gyeonggi-do, South Korea) and subsequently sequenced by standard Sanger methods in both directions using an ABI 3730xl DNA Analyzer (Hylabs, Rehovot, Israel).

The first successful isolated in ECE and adapted to tissue cultures BTV-9 was sent to FLI for confirmation of laboratory diagnosis. Using High Throughput Sequencing—Sequence-Independent Single-Primer-Amplification (HTS-SISPA) technology [45]—and the previously described procedure [46], double-sense (ds) cDNA of BTV-9 strain ISR-1763/3/19 was prepared and submitted to Eurofins Genomic (Ebersberg, Germany) for genome sequencing on Illumina platform. Obtained fast raw data were further processed using the Geneious Prime v2021.0.1 software (Biomatters Ltd., Auckland, New Zeeland) to construct complete genome sequence of the BTV-9 strain ISR-1763/3/19.

Whole genome sequencing (WGS) of the BTV-1 strain ISR-2050/19 was chosen for showing suitable concentration and RNA quality for WGS, compared to two other BTV-1 isolates. Sample preparation for WGS was performed at KVI, Israel. Extracted RNA was submitted to Genotypic Technology Pvt. Ltd., Bangalore, India. The whole procedure was described previously [34]. The resulting nucleotide (nt) sequences of the BTV-1 (NGS) and partial Sanger sequences of BTV-9 strains (2018 and 2020 years of collection) were assembled and nt sequences were aligned and pairwise compared by using Geneious version 9.0.5 (Biomatters, Auckland, New Zealand) and/or BioEdit programs (https://bioedit.software.informer.com/7.2/ (accessed on 12 February 2020)). Phylogenetic trees were constructed using the Mega X software [47]. For all phylogenetic trees, the maximum-likelihood method (ML) and the Tamura–Nei models were applied.

To gain insight into the phylogeny of the Israeli BTV-9 isolates, we performed BLAST and pairwise analyses of analogous genomic regions. Of note, due to the ~100% of nt identity by Seg-1, -2, -3, -4, -5, -6, and 10 of all eight BTV-9 strains used in the study, only the ISR-1763/3/19 strain was used for analyses of these segments.

## 3. Results

### 3.1. Clinical Signs in Affected Animals and Geographic Distribution of BTV-1

**Figure 1:**
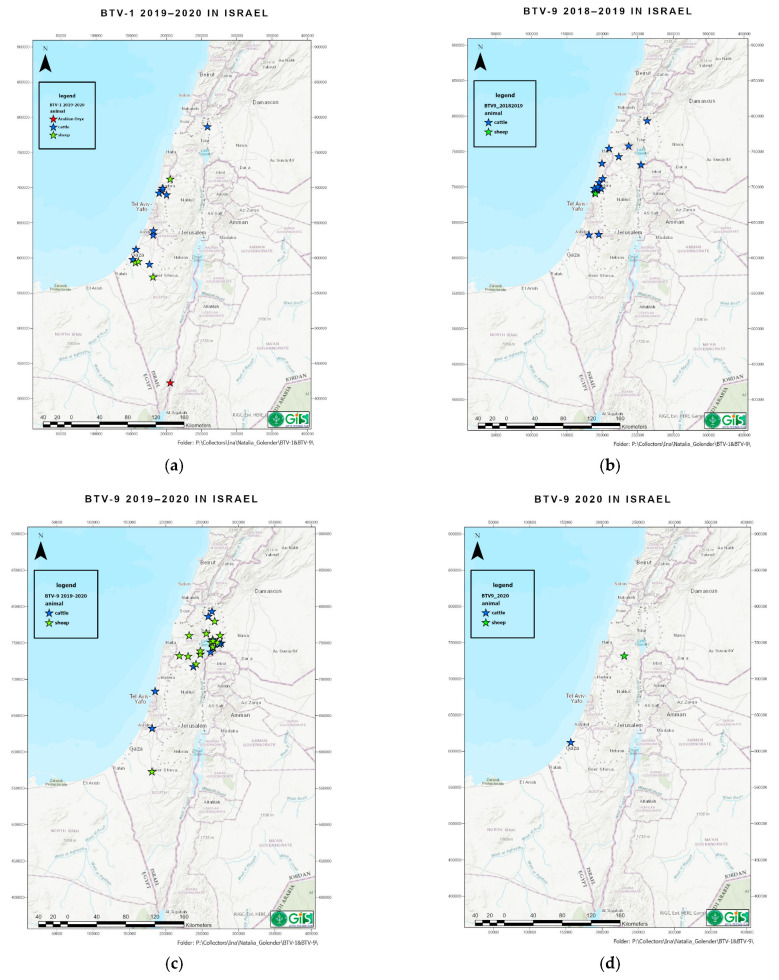
Distribution of BTV-1 and BTV-9 by seasons during 2018–2020. Stars show geographic settlements and type of animals where BTV was identified. (**a**) BTV-1 distribution during the end of 2019–beginning of 2020. Location of BTV-1-infected goats are not signed because BTV-1 was also identified in sheep in the same farm. (**b**) BTV-9 distribution during the end of 2018–beginning of 2019. (**c**) BTV-9 distribution during the end of 2019–beginning of 2020. (**d**) BTV-9 distribution during the end of 2020.

BTV-1 was detected in field samples collected from October 2019 until the beginning of February 2020. This fact allows us to presume that BTV-1 caused only a single-season outbreak. Taking in account that the virus was detected in all geographic areas of Israel (Figure 1) and many BTV-1 cases had an additional laboratory diagnosis (10/26 mixed, see below), we presume that many non-registered BTV-1 infections were mild or asymptomatic.

In only milk milking cows from five different farms, could a single BTV-1 infection be determined. These cows manifested milk reduction or/and fever and or/and recumbency, with one milking cow manifesting subcutaneous emphysema. One milking cow, which manifested fatigue, recumbency, and chills and one female 12-month calf showing recumbency, corneal opacity, and lacrimation were positive for BTV-1, -3, and-4. Another milking cow positive for BTV-1 was also positive in RT-PCR for bovine leukosis virus. A spleen sample from a young female calf (from the age-group of 2–6-month-old), which manifested, before death, bloody diarrhea, anorexia, inappetence, and conjunctivitis, was positive for BTV-1, -3, and -4. One milking cow that manifested a bloody nasal discharge was positive for BTV-1, -3, and -9. Several asymptomatic bulls were identified as BTV-1 positive, which blood samples were submitted for KVI for commercial purposes.

According to presented data, BTV-1 in cattle was associated with a mild clinical manifestation of the disease only, unless the cases of simultaneous additional pathogens were identified. However, we cannot determine decisively that these clinical signs were caused by BTV-1.

Sheep and goats identified as positive for BTV-1 in RT-qPCR manifested more severe clinical signs than cattle, which included lameness, oral mucosa hyperemia, and nasal bloody discharge. No positive BTV-1 aborted fetuses/placentas were identified. Two sheep from two closely located settlements were also positive for BTV-8, while in both cases BTV-1 was isolated [40]. The spleen from a dead 3-month-old lamb was positive for BTV-1 and -9. Blood samples from three goats were submitted due to heavy conjunctivitis, when all of them were positive for BTV: two were untyped, while one was identified as BTV-1 positive. One spleen from the dead Arabian Oryx was positive for BTV-1 and -3 (Table 2 and Table 3).

### 3.2. Clinical Signs in Affected Animals and Geographic Distribution of BTV-9

Since the number of BTV-9 detected animals was significantly higher than BTV-1, we describe only the most prominent observations. The first sample from BTV-9-affected cattle was submitted on 20 August 2018 from a cattle farm situated at the coastal central area of Israel, while the next positive case was registered on September 17th from cattle from the West Bank, located less than 30 km distance from the first place of BTV-9 detection. Afterward, half of the BTV-9 positive samples were sent from eight closely situated cattle and one sheep farm. From this geographic location, the virus spread to six farms located at northern part of Israel and two cattle farms situated southward from the first, as well as the most affected area, located in the central part of the country (about 70 km one from another). Notably, 24 of 25 positive domestic ruminants were cattle and one was sheep in 2018 (Figure 1b, Table 2). A total of 5 of the 24 samples from cattle were additionally positive for additional BTV serotypes (four cows were positive for BTV-3, and one for BTV-4), collected during October–November 2018.

In 2019, the first detection was from a sheep farm in the north of Israel at the end of July. In general, BTV-9 was registered in 14 sheep and 14 cattle farms, located from the Golan Height (North of Israel) to Negev desert (South of Israel), with most of the illness cases registered in the north of the country (Figure 1c). Only 7 in 92 BTV-9 positive field samples collected from symptomatic animals had a mixed infection with another BTV serotype (Table 2 and Table 3), which allow us to presume that most cases of illness were caused by BTV-9. Looking at the data in Table 2, it is obvious that BTV-9 was a dominant BT serotype both in cattle and sheep during 2019–2020 season. In 2020, only one female sheep from the north of the country and two milking cows from the south of the country, collected in November 2020, were positive for BTV-9 (Figure 1d).

Summarizing clinical signs in affected sheep flocks, ill animals demonstrated fatigue, facial edema, cyanosis of mucous membranes, serous or bloody nasal and oral discharges, eye discharge, abortions, fever, lameness or stiff gate, and acute death. High sheep mortality was seen in 2019, when 60% (9/15) of pan-BTV-positive dead sheep submitted to KVI were BTV-9 positive, and in at least two dead sheep pulmonary artery hemorrhages were seen during post-mortem examination. Two confirmed positive abortion sheep cases were also detected [35]. Clinical signs in milking cows included hypersalivation, fever, dyspnea, recumbency, milk reduction, and diarrhea.

### 3.3. BTV Detection by Pan-BTV RT-qPCR from Field Samples Collected in 2018–2020

In total, 754 animals out of 3111 tested ruminants (764 out of 3371 tested samples) were positive in Pan-BTV RT-qPCR during 2018–2020. Table 1 summarizes data on molecular detection of all BTV cases in field samples in 2018–2020. Results on virus isolation from the field samples collected in 2018 and 2019 were shown in Golender et al., 2021 [40], while detection of BTV-9 in aborted fetuses was previously presented in [35].

### 3.4. Virus Isolation (VI) from Field Collected in 2018–2020

Ninety-two BTV that belonged to serotypes 1, -2, -3, -4, -6, -9, -12, and -15 were isolated during 2018–2020. Full data on VI are presented in Table 1.

### 3.5. Serotype Specific RT-qPCR Result of Tested BTV-Positive Tested in 2018–2020

Depending on the results of virus isolation during the same season, Pan-BTV RT-qPCR positive samples were retrospectively tested for detection of the next serotypes (BTV-1, -3, -4, -8, -9 and -15). The comparison of primers/probes regions of published RT-qPCR systems for BTV-2, -6, and -12 [46] with the same genome regions of Israeli strains highlighted several mismatches, which may theoretically make these PCR systems less sensitive. For this reason, we decided not to use these PCR systems for serotyping of field samples. The results from these tests were presented in Table 2.

In addition to domestic animals, several spleen/lung samples collected from dead wild/zoo ruminants were positive in 2020. Thus, three Arabian oryxes were Pan-BTV positive: two of them, whose carcasses were collected in December 2020, were BTV-3 positive (Ct 33.96 and 32.28), while an additional one, collected in February 2020, mixed BTV-1 and -3 positive (Ct 32.28), all from the Yotvata (Southern region, Figure 1a). One spleen collected from the dead addax was low positive (Ct 38.4) and was not used for typing; two spleens from wild goats (Ct value 26.34, 33.51) were tested for five serotypes and found negative for all of them, and one spleen from a dead wild goat (Ct 33.0) was BTV-4-positive.

During 2018–2019 a small number of samples stayed untyped (18/213 (8.45%) in 2018; 16/193 (8.29%) in 2019). In contrast, there were about 40% untyped samples collected in 2020: 48 samples from cattle and 22 samples from sheep (samples from aborted fetuses are not included). During 2020 BTV-2, -6 and -12 were isolated. This fact allows us to presume that most BTV untyped samples probably belonged to these serotypes (Table 1 and Table 2).

Data on mixed infection on Israeli ruminants are shown in Table 3. In brief, no mixed infection was found in domestic goats, whose number in the total number of tested samples was small (Table 1). Most cases of mixed BTV infections were found in cattle. In sheep, mixed infection was observed in 2019 only. Mixed infection of BTV-2 with BTV-3 and BTV-2 with BTV-4 was detected during BTV-2 isolation in ECE and partial sequencing of segment (Seg) 2. Due to the absence of BTV-2 serotype-specific RT-qPCR, suitable for Israeli BTV-2, it is impossible to know both the real number of BTV-2 positive samples and the number of mixed BTV infection in field samples. Similarly, there are no data on real level on BTV-12 and BTV-6 infections of Israeli ruminants in recent years. Notably, all cases of mixed infection were registered at the end of arboviral season (October–February). Here, only a small number of mixed simultaneous virus isolations from molecularly detected mixed BT infections were successful. Therefore, we presume that if the majority of mixed BT can be explained by sequential BTV infections and long-lasting RNAmia, then less can probably be explained by simultaneous mix infections of several BTV serotypes. In addition to the detection of mixed BTV infections, combined BTV and epizootic hemorrhagic disease virus serotype 7 (EHDV-7) were also detected in cattle. Additionally, the combination of BTV-4 and EHDV, a non-typable BTV serotype, was also found together with EHDV.

### 3.6. Sequencing, Pairwise and BLAST Analyses of BTV-1 and BTV-9

All sequences obtained in this study were uploaded to the INSDC under the accession numbers listed in Table S2.

A BTV-1 ISR-2050/19 strain was completely sequenced. Full data about the strain are showed in Table S2. BLAST analysis based on comparison of nt sequences with global BTV is presented in Table 4. In brief, Israeli BTV-1 is a reassorted strain mostly with BTVs of African origin, while Seg-3 and -5 have close relationships with recent Israeli BTV-3 and BTV-6 strains. Seg-1 has the closest relationship with the Greek BTV-4 GRE2014/08 strain.

For the reasons of a prolonged period of identification of BTV-9 cases in Israel (2018–2020) and for a better understanding the dynamic of BTV-9 infections/explosions and outbreaks in Israel, it was decided that in addition to one completely sequenced BTV-9 ISR-1763/3/19 strain, a partial sequencing of all genome segments of some additional samples would be performed. For this purpose, one BTV-9 strain isolated in 2019, two BTV-9 positive available field samples collected in 2018, and two in three field samples collected in the end 2020 were sequenced using the Sanger method (Table S2). During analyses of the results, it was decided to sequence segments 7, -8 and -9 of three more BTV-9 isolated from samples collected from animals in different farms in 2019. Notably, attempts to isolate BTV-9 in 2018 and 2020 failed. All BTV-9 isolated in 2019 were closely related in all genome segments (identity 99.63–100%). For this reason, the only completely sequenced ISR-1763/3/19 strain was used as a representative strain of Israeli BTV-9 from 2019 (Table 5). Comparing strains from the whole period (2018–2020), pairwise analysis showed that all Israeli BTV-9 strains were very closely related to one another by Seg-1, -2, -3, -4, -5, -6, -9 and -10, having only point mutations. Interestingly, strains circulated in 2018 and 2020 were closely related one to another by all genome segments, while they had only 96.06–96.49% of nt identity with BTV-9s isolated in 2019 by Seg-7, and 94.12–94.19% nt identity by Seg-8. The strain ISR-2455/4/20 was used for pairwise and BLAST analyses as the representative strain of studied BTV-9 strains from the 2018–2020 years of collection. Seg-9 of the strain ISR-2758/1/20 is probably different from all other BTV-9 Israeli strains used in this study, and different in 2 nt out of 469 nt of the sequenced region, sharing 98.93% of nt identity with BTV-9 ISR-1763/3/19 strain (Figure S1g).

### 3.7. Phylogenetic Analysis of BTV-1 and BTV-9

The sequences of the Israeli ISR-2050/19 isolate were used for the phylogenetic analyses of BTV-1 serotype, and ISR-1763/3/19 participated as a representative strain for all phylogenetic trees of BTV-9. The strain ISR-2176/2/18 was the representative strain of BTV-9 from 2018 and the strain ISR-2455/4/20 from 2020 (for Seg-7 and -8 only, Figure S1a–h). In general, phylogenetic analysis of internal genes showed mostly the same results as BLAST analyses, which were presented in Table 4 and Table 5 and not described in details. Detailed data presented were (i) different from data presented in Table 4 and Table 5 and (ii) had several different results.

**Figure 2:**
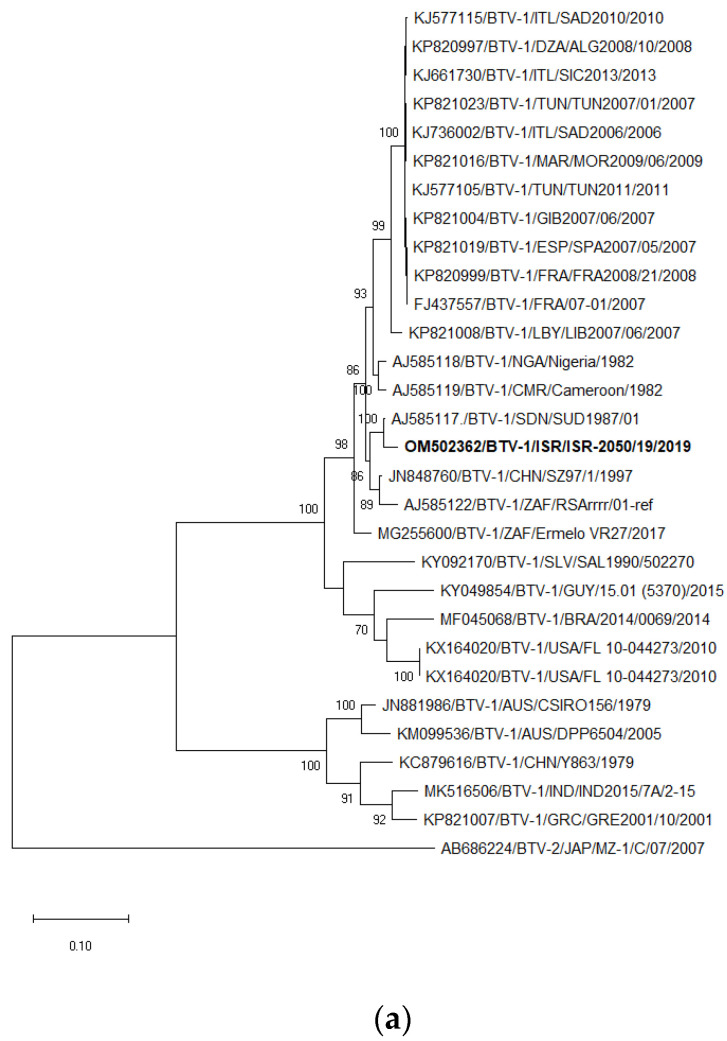

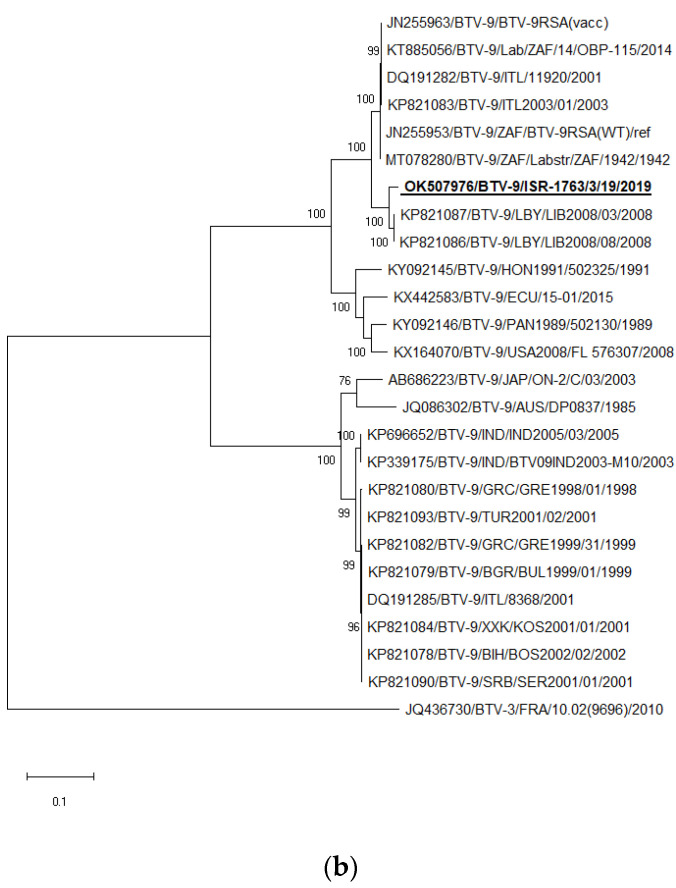
Phylogenetic tree of segment 2 of Israeli BTV-1 and BTV-9 strains isolated in 2019. (**a**) Phylogenetic tree of BTV-1 and global BTV-1 strains. BTV-2 was used as an outgroup. Israeli BTV-1 strain is shown in bold. (**b**) Phylogenetic tree of BTV-9 and global BTV-9 strains. BTV-3 was used as an outgroup. Israeli BTV-9 strain is shown in bold and is underlined. The phylogeny was inferred using the Maximum Likelihood method and Tamura–Nei model method. The percentage of replicate trees in which the associated taxa clustered together in the bootstrap test (1000 replicates) are shown next to the branches. Viruses were identified by accession number/serotype/location/isolate/year.

Segment 2. Similar to the BLAST analysis, according to phylogenetic analysis, Israeli BTV-1 clustered with the strain from Sudan SUD1987 (Figure 2a). Israeli BTV-9 clustered with Libyan LIB2008/03 and LIB2008/08 strains (Figure 2b).

**Figure 3:**
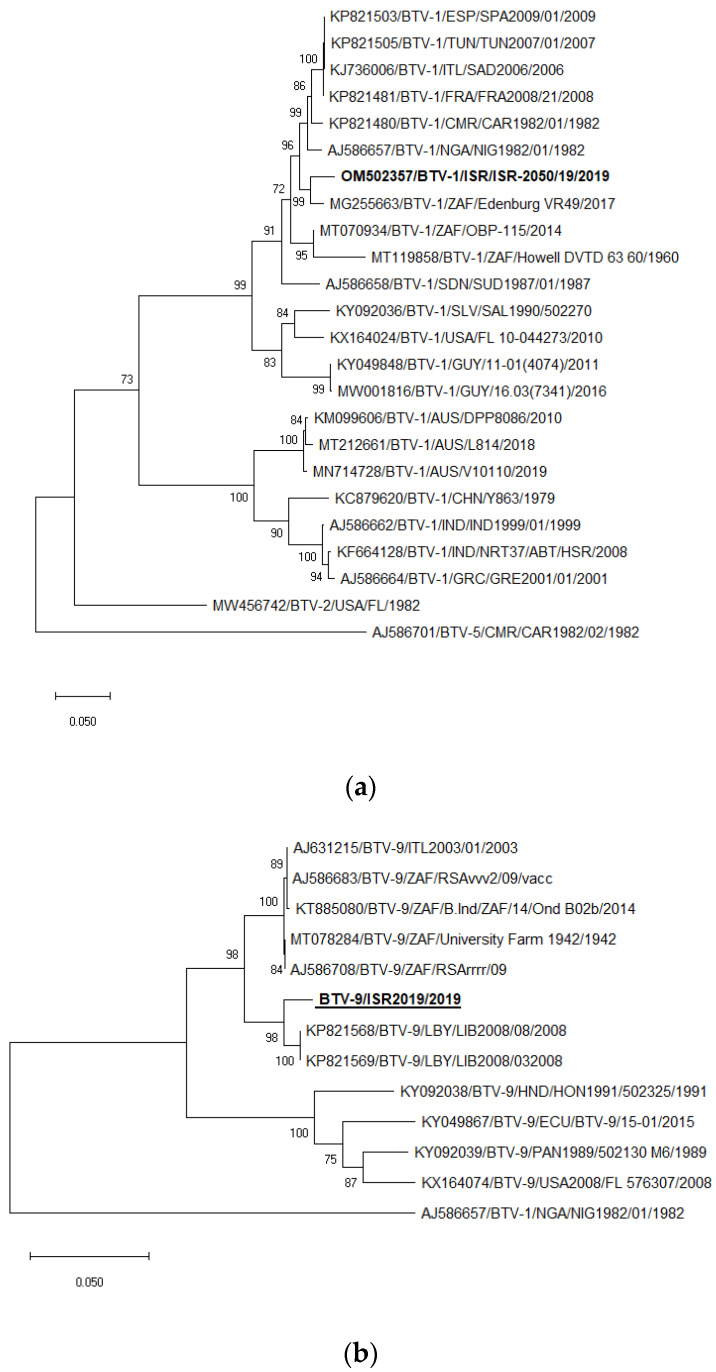
Phylogenetic tree of segment 6 of Israeli BTV-1 and BTV-9 strains isolated in 2019. (**a**) Phylogenetic tree of Israeli and global BTV-1 strains. BTV-5 was used as an outgroup. Israeli BTV-1 strain is shown in bold. (**b**) Phylogenetic tree of BTV-9 and global BTV-9 strains. BTV-1 was used as an outgroup. Israeli BTV-9 strain is shown in bold and underlined. The phylogeny was inferred using the Maximum Likelihood method and Tamura–Nei model method. The percentage of replicate trees in which the associated taxa clustered together in the bootstrap test (1000 replicates) are shown next to the branches. Viruses were identified by accession number/serotype/location/isolate/year.

Segment 6. Similar to the BLAST analysis, phylogenetic analysis revealed that Israeli BTV-1 clustered with the South African BTV-1 VR49 strain isolated in 2017 (Figure 3a). Phylogenetic analysis showed the close relationship of Israeli BTV-9 and Libyan BTV-9 strains (LYB2008/03 and LYB2008/08), similar to the BLAST analysis (Figure 3b).

Segment 5. The Israeli BTV-9 clustered with untyped BTV ISR-2237/18, which was detected in the brain tissue of a dead calf, manifested neural signs before death and was submitted to KVI for pathogen identification, where Shuni virus was identified [34] (Figure S1d).

Segment 7. The Israeli BTV-9 strains isolated in 2019 clustered with Israeli BTV-4 strains isolated in 2013 and 2017 were also similar to the results of BLAST analysis, while Israeli BTV-9 strains identified in 2018 and 2020 clustered with Libyan BTV-9 LYB2008/03 and LYB2008/08 strains isolated in 2008 (Figure S1e).

Segment 8. The BTV-9 strains isolated in 2019 clustered with Israeli BTV-2 and BTV-4 strains isolated in 2017. BTV-9 identified in field samples collected in 2018 and 2020 clustered with BTV-4 strain SUD1983/01 (Figure S1f).

## 4. Discussion

In Israel BTV-1 was recognized in field samples from October 2019 until February 2020 in a small number of field samples, which were collected all over the country in cattle, sheep, and one Arabian oryx. Many cases of mixed infections allowed presuming that in the majority of the registered cases BTV-1 was an occasional finding, causing mild illness in susceptible animals.

The BTV-9 positive samples were identified retrospectively in samples collected in August 2018 in coastal central area of Israel and in the West Bank, further spreading mostly in cattle farms from the north of Israel, but being registered southward also. In 2019, it reappeared and spread in the northern part of Israel among mostly sheep farms, while further it also was found in farms located in the central and southern parts of Israel. The last very few cases of BTV-9 were registered in November 2020 in one geographic place in the north and in one in the south of Israel. Observed clinical manifestation of the disease in sheep included fatigue, facial edema, cyanosis of mucous membranes, serous or bloody nasal and oral discharges, eye discharges, abortions, fever, lameness or stiff gate, and acute death with/without classical BT pathologic findings. Contrarily, BTV-9-infected milking cows manifested hypersalivation, fever, dyspnea, recumbency, milk reduction, and diarrhea.

Comparing the Israeli BTV-1 strain to the global BTV-1 strains, the broad identity of the VP2 gene with the BTV-1 strain from Sudan (strain SUD1987/01; 98.00%) and the broad identity of the VP5 gene with the BTV-1 strain from South Africa (nt identity 96.67%) suggests that the Israeli BTV-1 strain has not yet been identified, or that it appeared as a result of a reassortment between these African BTV-1 strains. Looking on the BTV-9, a very close identity between both outer protein coding genes to Libyan BTV-9 strain isolated in 2008 and Seg-7 of BTV-9 from 2018 and 2020 points out a common ancestor of Israeli and Libyan BTV-9s.

Sequencing of the BTV-1 and the BTV-9 showed that both BTV-1 and BTV-9 have the same ancestor for the segment 4 (Tunisian BTV-3 TUN2016/Zarzis strain), probably pointing out the same area of co-circulation of all these three strains. Notably, Israeli BTV-1 was reassorted with local Israeli strains by only two segments (segments 3 and 5). The BTV-9 strains identified in 2018 and 2020, which were not possible to isolate, have three segments reassorted with local strains (segments 1, 3, and 5). In contrast to BTV-9 strains 2018 and 2020, the BTV-9 strain from 2019 was reassorted by five segments with local strains (segments 1, 3, 5, 7, and 8), being identified in an absolute majority of submitted symptomatic cases from domestic ruminants and in aborted sheep fetuses that year. Basing on this data, we can presume that for switching from “low pathogenic” to more “high pathogenic”, BTV-9 had to reassort with local endemic BTV-4 and BTV-2 strains. For the reason of absence samples from healthy animals, evaluation of the role of any BTV serotype/strain in subclinical/asymptomatic cases in Israel was impossible. It can be explained by having better infection ability of local *Culicoides* spp. due to VP7 role for *Culicoides* infection [48], or/and better transmission and infection. It is difficult to evaluate the role of these segments in absence of successful virus isolation. As it was already seen before, not every novel registered BTV strain caused an outbreak. Thus, the appearance of the “Nigerian” BTV-8 did not cause the successful spread of the virus among Israeli ruminants, with only two cases of the strain being registered in 2019 [40] and no BTV-8 cases being identified among tested pan-BTV positive field samples and virus isolation in 2020.

Summarizing all studied data on both Israeli BTV-1 and BTV-9, the dynamic of the spread and the type of the outbreaks were different. Thus, the BTV-1 caused a single-season outbreak, spreading all over the country, infecting both domestic and wild ruminants, but probably being the cause of mild illness in ruminants. This conclusion is based on summarized data of a small number of registered cases/submitted field samples for the laboratory diagnosis, and mild illness and the small number/percentage of BTV-1 genome detections in organs of the dead animals. The virus could spread all over the country for less than three months (October–December 2019) without substantial clinical pictures in the field. Due to the absence of successful virus isolations, identification of BTV-1 cases by RT-qPCR in January-February 2020 can be explained by prolonged RNAmia, but not by “fresh” infections.

Opposite to BTV-1, BTV-9 caused serious outbreak in 2019 and was the main BTV strain responsible for substantial sheep mortality and cattle morbidity in that season. Surprisingly, BTV-9 were retrospectively recognized in field samples collected in 2018. The same situation, when considering that a small percent of positive cases were registered a year before a heavy outbreak, was observed on example with BTV-3. Thus, several years were needed for the successful spread of the BTV-3 from the southern part of Israel [39] northward. Similarly, BTV-8 reappeared in 2015, causing a heavy outbreak in 2016 in Israel and even in Cyprus [40]. The same situation was seen with an outbreak caused by BTV-15. It reappeared in 2016. In 2017, the proportion of BTV-15 positive cases in total BTV positive cases increased comparing to 2016, causing a heavy outbreak in cattle in the following year, 2018 (serotyping of positive samples 2018, Table 2) [40]. In a similar manner to the aforementioned situations of BTV spread in Israel, in 2006, BTV-8 was first registered in Europe in an area between the Netherlands, Belgium, and northern Germany, and then spread throughout the continent and the United Kingdom, causing a high mortality in naive sheep flocks and clinical disease in cattle in 2007–2009 [49,50]. Such “delay” from the first registration until the outbreak among susceptible ruminant population on large territory might be linked with the adaptation of BTV strains to the local condition.

Serotyping of Israeli BTV positive in Pan-BTV RT-qPCR samples is based on using published in-house real-time RT-PCR systems. Unfortunately, some published RT-qPCR system [44], which were developed about 10 years ago, cannot be used for sample serotyping due to the existence of many mismatches between currently circulating BTV strains in primer/probe regions of the systems, as BTV-2, -6, -12. This fact complicates the quick identification of circulating viruses. Consequently, the adaptation and optimization of molecular diagnostic methods for the easy characterization and serotyping of BTV in field samples is necessary. The prerequisite for the developing of adapted molecular assays is the generation of useful sequence data, whereby the definition of whole-genome data is preferred.

In summary, a monitoring or surveillance network for the emergence of new strains/serotypes and the re-emergence of existing strains/serotypes would be useful for the development of effective prevention measures. Such a procedure was carried out and was very successful in detecting circulating influenza virus strains, including new variants [51]. Additionally, the establishment of appropriate surveillance programs for BTV would allow the selection and early development of suitable vaccines for the next epidemic season.

## Figures and Tables

**Table 1:** Pan-BTV RT-qPCR and BTV isolation from different kinds of domestic and wild/zoo ill or dead animals tested from 2018–2020.

Species		Cattle			Sheep			Goat			Wild Ruminants					
Year/Organ		w.b.	s/l	a.f	w.b.	s/l	a.f	w.b.	s/l	a.f	w.b.	s/l	a.f	Total	Total VI	Source
2018	№ of tested samples	629	18	53	72	32	198	8	21	14	4	13	3	1065		[35,40] *
	№ of tested animals	629	18	51	72	32	156	8	21	14	4	13	3	1021		
	№ of pos. samples	217	6	1	36	0	6	3	2	0	0	0	0	271		
	№ of pos. animals	217	6	1	36	0	6	3	2	0	0	0	0	271		
	№ of isolated BTV-2	1													1	
	№ of isolated BTV-3	1			6			1							8	
	№ of isolated BTV-4	1			9										10	
	№ of isolated BTV-6				2										2	
	№ of isolated BTV-15	9													9	
2019	№ of tested samples	490	45	112	109	51	140	7	5	17	23	26	5	1030		[35,40] *
	№ of tested animals	490	45	87	109	51	98	7	5	13	23	26	5	959		
	№ of pos. samples	106	8	1	52	17	9	4	0	0	0	0	0	197		
	№ of pos. animals	106	8	1	52	17	7	4	0	0	0	0	0	195		
	№ of isolated BTV-1	3			2										5	
	№ of isolated BTV-3	5	1		2										8	
	№ of isolated BTV-4	1			6										7	
	№ of isolated BTV-9	7			17	1									25	
2020	№ of tested samples	560	86	93	155	76	186	2	22	28	3	59	6	1276		current
	№ of tested animals	560	86	59	155	72	100	2	20	18	3	51	5	1131		study
	№ of pos. samples	169	10	1	72	12	22	0	2	1	0	7	0	296		
	№ of pos. animals	169	10	1	72	8	18	0	2	1	0	7	0	288		
	№ of isolated BTV-2	1			3										4	
	№ of isolated BTV-3	1			5		2								8	
	№ of isolated BTV-4				3										3	
	№ of isolated BTV-6				1										1	
	№ of isolated BTV-12	1													1	
total	№ of tested samples	1679	149	258	336	159	524	17	48	59	30	98	14	3371		current
	№ of tested animals	1679	149	197	336	155	354	17	46	45	30	90	13	3111		study
	№ of pos. samples	492	24	3	160	29	37	7	4	1	0	7	0	764		
	№ of pos. animals	492	24	3	160	25	31	7	4	1	0	7	0	754		
	№ of isolated BTV-1	3			2									5	5	
	№ of isolated BTV-2	2			3									5	5	
	№ of isolated BTV-3	7	1		13		2	1						24	24	
	№ of isolated BTV-4	2			18									20	20	
	№ of isolated BTV-6				3									3	3	
	№ of isolated BTV-9	7			17	1								25	25	
	№ of isolated BTV-12	1												1	1	
	№ of isolated BTV-15	9												9	9	

w.b.—whole blood samples; s/l—spleen or lung samples; a.f—aborted fetus; VI—virus isolation. *—in [40] the number of aborted cases (animals) was presented as a number of tested samples in 2018–2019, and [35] presented the complete data on a total number of tested abortion cases and tested samples.

**Table 2:** Positive samples from domestic ruminants tested in serotype-specific qPCR during 2018–2020.

			Cattle			Sheep			Goat			
	RT-qPCR		w.b.	s/l	a.f	w.b.	s/l	a.f	w.b.	s/l	a.f	Total
2018	BTV-3	№ of tested samples	155	5	1	31	0	16	3	2	0	213
		№ of positive	13	2	0	8	0	0	1	1	0	25
		% of positive	8.4	33.3	0	25.8	0	0	33.3	50	0	11.7
	BTV-4	№ of tested samples	155	5	1	31	0	16	3	2	0	213
		№ of positive	11	2	1	17	0	1	1	0	0	33
		% of positive	7.1	33.3	100	54.8	0	6.2	33.3	0	0	15.4
	BTV-8	№ of tested samples	155	5	1	31	0	16	3	2	0	213
		№ of positive	0	0	0	0	0	1 *	0	0	0	1 *
		% of positive	0	0	0	0	0	6.3 *	0	0	0	0.5 *
	BTV-9	№ of tested samples	155	6	1	31	0	16	3	2	0	213
		№ of positive	22	0	0	1	0	1	0	0	0	24
		% of positive	14.2	0	0	0	0	6.3	0	0	0	11.27
	BTV-15	№ of tested samples	155	6	1	31	0	16	3	2	0	213
		№ of positive	111	3	0	2	0	1	1	0	0	118
		% of positive	71.6	50	0	6.4	0	6.3	33.3	0	0	55.4
2019	BTV-1	№ of tested samples	101	8	1	52	15	9	4	0	1	191
		№ of positive	13	1	0	3	1	0	1	0	0	19
		% of positive	12.9	37.3	0	3.9	13.3	0	25	0	0	9.9
	BTV-3	№ of tested samples	102	8	1	52	15	9	4	0	1	192
		№ of positive	19	2	0	16	3	1	0	0	0	41
		% of positive	18.6	25	0	30.8	20	11.1	0	0	0	21.4
	BTV-4	№ of tested samples	102	8	1	52	15	9	4	0	1	192
		№ of positive	12	3	0	17	2	0	1	0	0	35
		% of positive	11.8	37.5	0	32.7	13.3	0	25	0	0	18.2
	BTV-8	№ of tested samples	103	8	1	52	15	9	4	0	1	193
		№ of positive	1	0	0	2	0	0	0	0	0	3
		% of positive	0.97	0	0	3.9	0	0	0	0	0	1.6
	BTV-9	№ of tested	102	8	1	52	15	9	4	0	1	192
		№ of positive	60	1	0	18	9	2	0	0	0	91
		% of positive	58.8	12.5	0	34.6	60	22.2	0	0	0	47.4
	BTV-15	№ of tested samples	103	8	1	52	15	9	4	0	1	193
		№ of positive	0	1	0	0	0	0	0	0	0	1
		% of positive	0	12.5	0	0	0	0	0	0	0	0.52
2020	BTV-1	№ of tested samples	142	5	1	69	7	11	0	1	0	236
		№ of positive	6	0	0	0	0	0	0	0	0	6
		% of positive	4.22	0	0	0	0	0	0	0	0	2.54
	BTV-3	№ of tested samples	142	5	1	69	7	11	0	1	0	236
		№ of positive	63	3	0	37	6	7	0	1	0	117
		% of positive	43.4	60	1	53.6	82.7	63.6	0	100	0	49.6
	BTV-4	№ of tested samples	142	5	1	69	7	11	0	1	0	236
		№ of positive	19	1	0	9	0	0	0	0	0	29
		% of positive	13.4	20	0	13.04	0	0	0	0	0	12.3
	BTV-8	№ of tested samples	142	5	1	69	7	11	0	1	0	236
		№ of positive	0	0	0	0	0	0	0	0	0	0
		% of positive	0	0	0	0	0	0	0	0	0	0
	BTV-9	№ of tested samples	142	5	1	69	7	11	0	1	0	236
		№ of positive	10	1	1	1	0	0	0	0	0	13
		% of positive	7.1	20	100	1.5	0	0	0	0	0	5.5

w.b.—whole blood, s/l—spleen/ lung; a.f—aborted fetus or newborn animal. Data on serotyping of positive samples collected during 2018 and 2019 was partially published [35,40]. *—the sample from this aborted sheep fetus had 100% nt identity NS1 sequenced region of BTV-8, while no data on Seg-2 is available [40].

**Table 3:** Mixed BTV and EHDV-7 cases identified by serotype specific RT-qPCR or/and partial sequencing of segment 2 of isolated viruses during 2018–2020.

Year	Species	Mixed BTV serotypes														
		1, 3	1, 3, 4	1, 3, 9	1, 8	1, 9	2, 3	2, 4	3, 4	3, 6	3, 9	3, 15	4, 9	4, 15	4, EHDV	UT, EHDV
2018	cattle								1		4	2	1	3		
2019	cattle	1	3						1		1		2			
	sheep				2	1			2	1						
2020	cattle	1		1			1	1	5		2				1	3
	Arabian Oryx	1														

UT-untyped.

**Table 4:** BLAST analysis of Israeli BTV-1 ISR-2050/19 isolate with global BTV strains.

Segment	Identity (%)	Accession Number/Serotype/Strain/Year	Country of Isolation
1	96.86	MT879211/BTV-4/GRE2014/08/2014	Greece
2	98.00	KP821022/BTV-1/SUD1987/01/1987	Sudan
3	98.12	MN200304/BTV-3/ISR-2210/18/2018	Israel
4	98.53	MF124295/BTV-3/TUN2016/Zarzis/2016	Tunisia
5	98.40	MH383093/BTV-6/ISR-2095/3/17/2017	Israel
6	96.67	MG255663/BTV-1/VR49_2017/2017	South Africa
7	98.16	KP821612/BTV-1/LIB2007/06/2007	Libya
8	97.00	MG255457/BTV-5/01012015/2015	South Africa
9	98.66	MF124300/BTV-3/TUN2016/Zarzis/2016	Tunisia
10	99.15	MG255548/BTV-3/VR11_2017/2017	South Africa

**Table 5:** BLAST analysis of Israeli BTV-9 ISR-1763/3/19 stain with global BTV strains.

Segment	Identity (%)	Accession Number/Serotype/Strain/Year	Country of Isolation
1	99.59	MG344990/BTV-3/ISR-2153/16/2016	Israel
2	99.82	KP821086/BTV-9/LIB2008/08/2008	Libya
3	99.19	MH383091/BTV-6/ISR-2095/3/17/2017	Israel
4	98.47	MF124295/BTV-3/TUN2016/Zarzis/2016	Tunisia
5	99.51	ON087703/BTV/ISR-2237/18/2018	Israel
6	98.14	KP821568/BTV-9/LIB2008/08/2008	Libya
7	99.80	MK893198/BTV-4/ISR-1899/13/2013	Israel
8	99.90	MK893195/BTV-4/ISR-1779/3/17/2017	Israel
9	96.53	MG255454/BTV-5/01012015/2015	South Africa
10	98.21	KP196612/BTV/BT 57/08/2008	South Africa

## Data Availability

Not applicable.

## Supplementary Materials

The following supporting information can be downloaded at: https://www.mdpi.com/article/10.3390/microorganisms11020366/s1, Figure S1: Phylogenetic threes genome segments coding internal genes of BTV-1 and BTV-9 Israeli strains with global BTV strains. (a) segment 1; (b) segment 3; (c) segment 4; (d) segment 5; (e) segment 7; (f) segment 8; (g) segment 9; (h) segment 10. Table S1: List of primers used for partial sequencing of Israeli bluetongue virus serotype 9; Table S2: List of sequenced Israeli BTV strains used for the present study.
