# Supplementary material for: Comparative Molecular and Epidemiological Analyses of Israeli Bluetongue Viruses Serotype 1 and 9 Causing Outbreaks in 2018–2020
Source: Microorganisms. 2023 Feb 1;11(2):366. doi: 10.3390/microorganisms11020366 (PMC9966015; doi:10.3390/microorganisms11020366)
Supplement: Supplementary file 1 [file microorganisms-11-00366-s001.zip › microorganisms-2113390-SI.pdf]

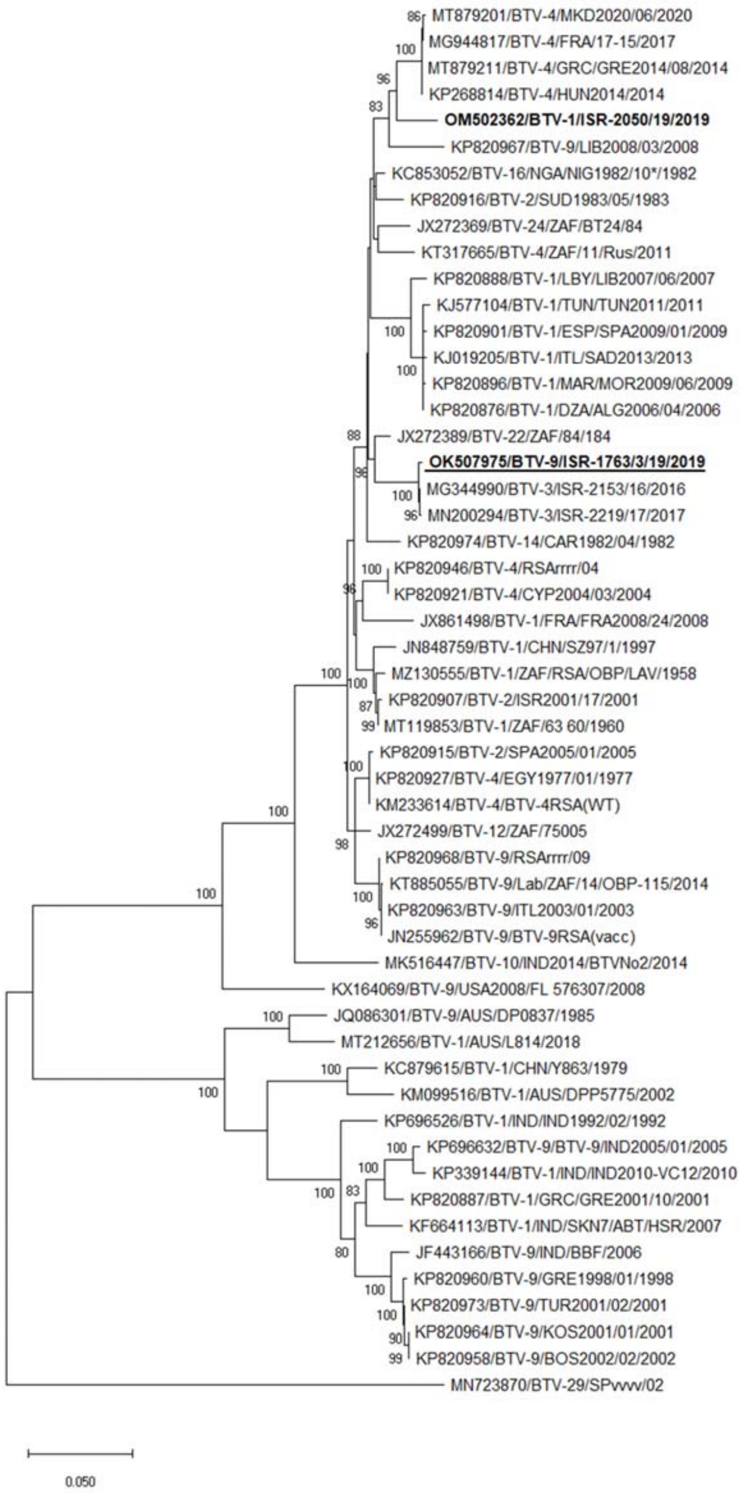
MT879201/BTV-4/MKD2020/06/2020
MG944817/BTV-4/FRA/17-15/2017
MT879211/BTV-4/GRC/GRE2014/08/2014
KP268814/BTV-4/HUN2014/2014
OM502362/BTV-1/ISR-2050/19/2019
KP820967/BTV-9/LIB2008/03/2008
KC853052/BTV-16/NGA/NIG1982/10*/1982
KP820916/BTV-2/SUD1983/05/1983
JX272369/BTV-24/ZAF/BT24/84
KT317665/BTV-4/ZAF/11/Rus/2011
KP820888/BTV-1/LBY/LIB2007/06/2007
KJ577104/BTV-1/TUN/TUN2011/2011
KP820901/BTV-1/ESP/SPA2009/01/2009
KJ019205/BTV-1/ITL/SAD2013/2013
KP820896/BTV-1/MAR/MOR2009/06/2009
KP820876/BTV-1/DZA/ALG2006/04/2006
JX272389/BTV-22/ZAF/84/184
OK507975/BTV-9/ISR-1763/3/19/2019
MG344990/BTV-3/ISR-2153/16/2016
MN200294/BTV-3/ISR-2219/17/2017
KP820974/BTV-14/CAR1982/04/1982
KP820946/BTV-4/RSArrrr/04
KP820921/BTV-4/CYP2004/03/2004
JX861498/BTV-1/FRA/FRA2008/24/2008
JN848759/BTV-1/CHN/SZ97/1/1997
MZ130555/BTV-1/ZAF/RSA/OBP/LAV/1958
KP820907/BTV-2/ISR2001/17/2001
MT119853/BTV-1/ZAF/63 60/1960
KP820915/BTV-2/SPA2005/01/2005
KP820927/BTV-4/EGY1977/01/1977
KM233614/BTV-4/BTV-4RSA(WT)
JX272499/BTV-12/ZAF/75005
KP820968/BTV-9/RSArrrr/09
KT885055/BTV-9/Lab/ZAF/14/OBP-115/2014
KP820963/BTV-9/ITL2003/01/2003
JN255962/BTV-9/BTV-9RSA(vacc)
MK516447/BTV-10/IND2014/BTVNo2/2014
KX164069/BTV-9/USA2008/FL 576307/2008
JQ086301/BTV-9/AUS/DP0837/1985
MT212656/BTV-1/AUS/L814/2018
KC879615/BTV-1/CHN/Y863/1979
KM099516/BTV-1/AUS/DPP5775/2002
KP696526/BTV-1/IND/IND1992/02/1992
KP696632/BTV-9/BTV-9/IND2005/01/2005
KP339144/BTV-1/IND/IND2010-VC12/2010
KP820887/BTV-1/GRC/GRE2001/10/2001
KF664113/BTV-1/IND/SKN7/ABT/HSR/2007
JF443166/BTV-9/IND/BBF/2006
KP820960/BTV-9/GRE1998/01/1998
KP820973/BTV-9/TUR2001/02/2001
KP820964/BTV-9/KOS2001/01/2001
KP820958/BTV-9/BOS2002/02/2002
MN723870/BTV-29/SPvvvv/02
0.050

(a)

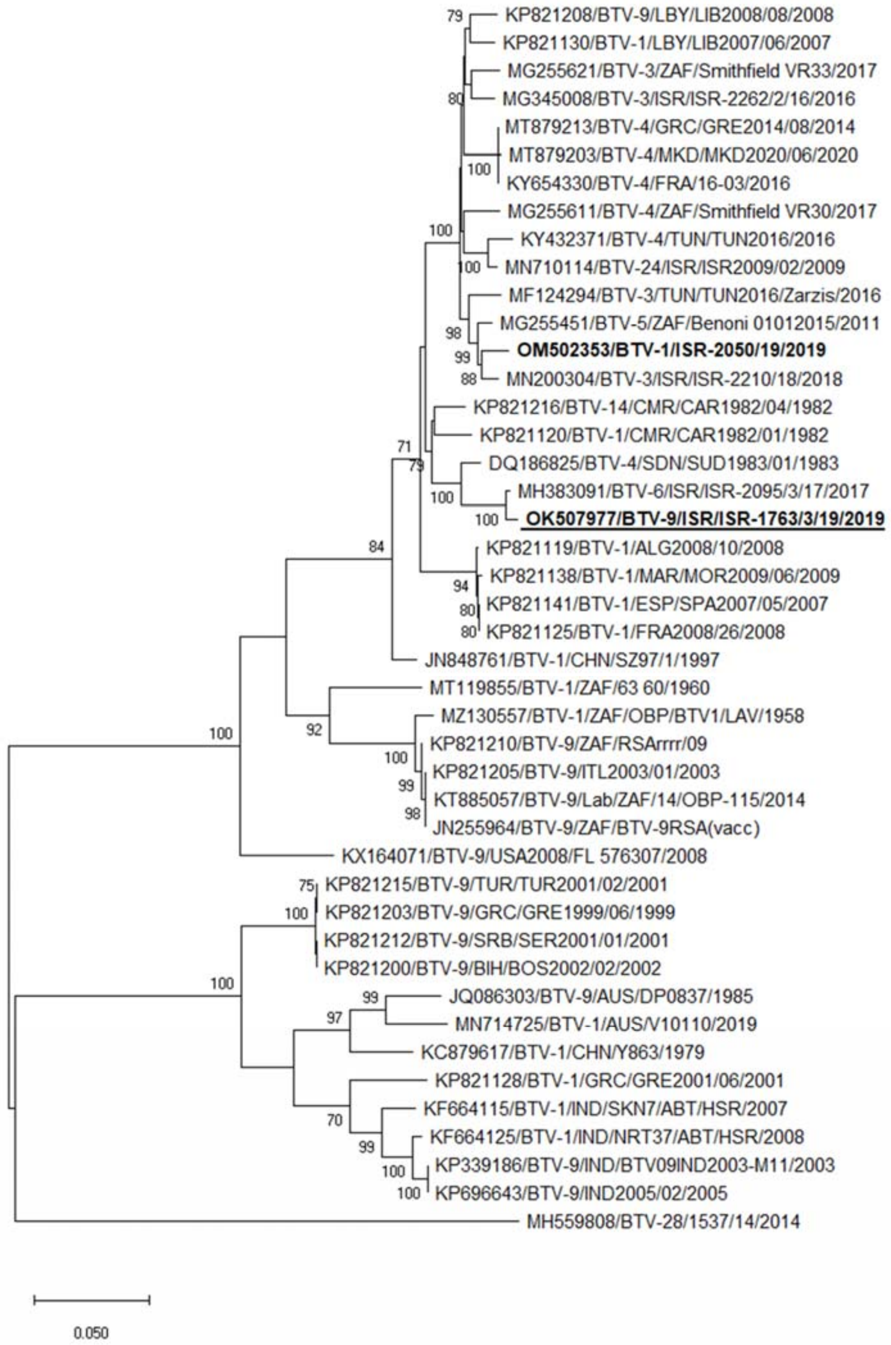
KP821208/BTV-9/LBY/LIB2008/08/2008
KP821130/BTV-1/LBY/LIB2007/06/2007
MG255621/BTV-3/ZAF/Smithfield VR33/2017
MG345008/BTV-3/ISR/ISR-2262/2/16/2016
MT879213/BTV-4/GRC/GRE2014/08/2014
MT879203/BTV-4/MKD/MKD2020/06/2020
KY654330/BTV-4/FRA/16-03/2016
MG255611/BTV-4/ZAF/Smithfield VR30/2017
KY432371/BTV-4/TUN/TUN2016/2016
MN710114/BTV-24/ISR/ISR2009/02/2009
MF124294/BTV-3/TUN/TUN2016/Zarzis/2016
MG255451/BTV-5/ZAF/Benoni 01012015/2011
OM502353/BTV-1/ISR-2050/19/2019
MN200304/BTV-3/ISR/ISR-2210/18/2018
KP821216/BTV-14/CMR/CAR1982/04/1982
KP821120/BTV-1/CMR/CAR1982/01/1982
DQ186825/BTV-4/SDN/SUD1983/01/1983
MH383091/BTV-6/ISR/ISR-2095/3/17/2017
OK507977/BTV-9/ISR/ISR-1763/3/19/2019
KP821119/BTV-1/ALG2008/10/2008
KP821138/BTV-1/MAR/MOR2009/06/2009
KP821141/BTV-1/ESP/SPA2007/05/2007
KP821125/BTV-1/FRA2008/26/2008
JN848761/BTV-1/CHN/SZ97/1/1997
MT119855/BTV-1/ZAF/63 60/1960
MZ130557/BTV-1/ZAF/OBP/BTV1/LAV/1958
KP821210/BTV-9/ZAF/RSArrrr/09
KP821205/BTV-9/ITL2003/01/2003
KT885057/BTV-9/Lab/ZAF/14/OBP-115/2014
JN255964/BTV-9/ZAF/BTV-9RSA(vacc)
KX164071/BTV-9/USA2008/FL 576307/2008
KP821215/BTV-9/TUR/TUR2001/02/2001
KP821203/BTV-9/GRC/GRE1999/06/1999
KP821212/BTV-9/SRB/SER2001/01/2001
KP821200/BTV-9/BIH/BOS2002/02/2002
JQ086303/BTV-9/AUS/DP0837/1985
MN714725/BTV-1/AUS/V10110/2019
KC879617/BTV-1/CHN/Y863/1979
KP821128/BTV-1/GRC/GRE2001/06/2001
KF664115/BTV-1/IND/SKN7/ABT/HSR/2007
KF664125/BTV-1/IND/NRT37/ABT/HSR/2008
KP339186/BTV-9/IND/BTV09IND2003-M11/2003
KP696643/BTV-9/IND2005/02/2005
MH559808/BTV-28/1537/14/2014
0.050

(b)

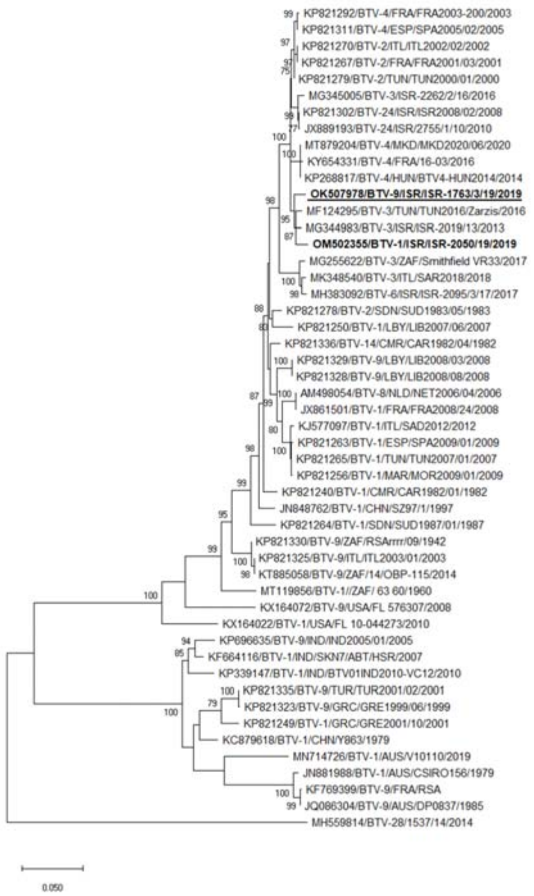
KP821292/BTV-4/FRA/FRA2003-200/2003
KP821311/BTV-4/ESP/SPA2005/02/2005
KP821270/BTV-2/ITL/ITL2002/02/2002
KP821267/BTV-2/FRA/FRA2001/03/2001
KP821279/BTV-2/TUN/TUN2000/01/2000
MG345005/BTV-3/ISR-2262/2/16/2016
KP821302/BTV-24/ISR/ISR2008/02/2008
JX889193/BTV-24/ISR/2755/1/10/2010
MT879204/BTV-4/MKD/MKD2020/06/2020
KY654331/BTV-4/FRA/16-03/2016
KP268817/BTV-4/HUN/BTV4-HUN2014/2014
OK507978/BTV-9/ISR/ISR-1763/3/19/2019
MF124295/BTV-3/TUN/TUN2016/Zarzis/2016
MG344983/BTV-3/ISR/ISR-2019/13/2013
OM502355/BTV-1/ISR/ISR-2050/19/2019
MG255622/BTV-3/ZAF/Smithfield VR33/2017
MK348540/BTV-3/ITL/SAR2018/2018
MH383092/BTV-6/ISR/ISR-2095/3/17/2017
KP821278/BTV-2/SDN/SUD1983/05/1983
KP821250/BTV-1/LBY/LIB2007/06/2007
KP821336/BTV-14/CMR/CAR1982/04/1982
KP821329/BTV-9/LBY/LIB2008/03/2008
KP821328/BTV-9/LBY/LIB2008/08/2008
AM498054/BTV-8/NLD/NET2006/04/2006
JX861501/BTV-1/FRA/FRA2008/24/2008
KJ577097/BTV-1/ITL/SAD2012/2012
KP821263/BTV-1/ESP/SPA2009/01/2009
KP821265/BTV-1/TUN/TUN2007/01/2007
KP821256/BTV-1/MAR/MOR2009/01/2009
KP821240/BTV-1/CMR/CAR1982/01/1982
JN848762/BTV-1/CHN/SZ97/1/1997
KP821264/BTV-1/SDN/SUD1987/01/1987
KP821330/BTV-9/ZAF/RSArrrr/09/1942
KP821325/BTV-9/ITL/ITL2003/01/2003
KT885058/BTV-9/ZAF/14/OBP-115/2014
MT119856/BTV-1//ZAF/ 63 60/1960
KX164072/BTV-9/USA/FL 576307/2008
KX164022/BTV-1/USA/FL 10-044273/2010
KP696635/BTV-9/IND/IND2005/01/2005
KF664116/BTV-1/IND/SKN7/ABT/HSR/2007
KP339147/BTV-1/IND/BTV01IND2010-VC12/2010
KP821335/BTV-9/TUR/TUR2001/02/2001
KP821323/BTV-9/GRC/GRE1999/06/1999
KP821249/BTV-1/GRC/GRE2001/10/2001
KC879618/BTV-1/CHN/Y863/1979
MN714726/BTV-1/AUS/V10110/2019
JN881988/BTV-1/AUS/CSIRO156/1979
KF769399/BTV-9/FRA/RSA
JQ086304/BTV-9/AUS/DP0837/1985
MH559814/BTV-28/1537/14/2014
0.050

(c)

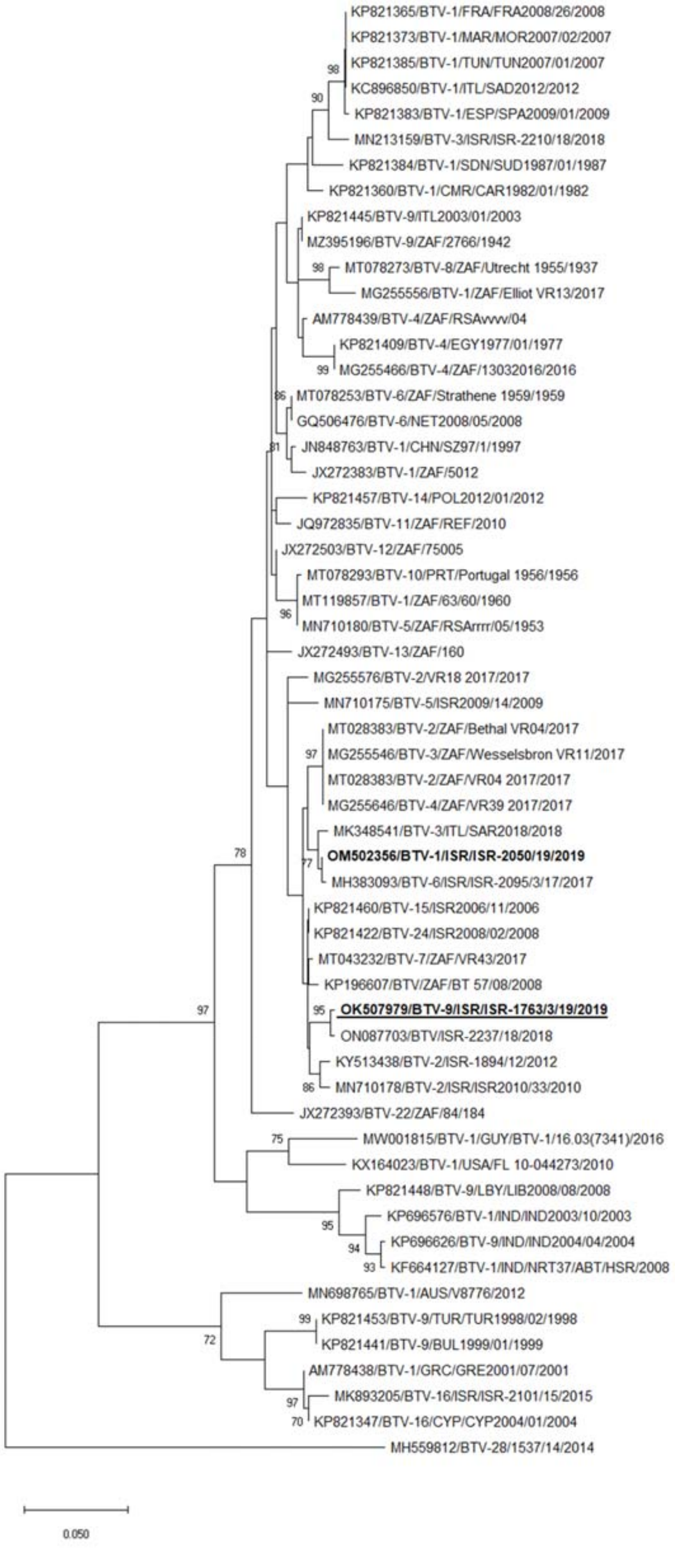
KP821365/BTV-1/FRA/FRA2008/26/2008
KP821373/BTV-1/MAR/MOR2007/02/2007
98
KP821385/BTV-1/TUN/TUN2007/01/2007
90
KC896850/BTV-1/ITL/SAD2012/2012
KP821383/BTV-1/ESP/SPA2009/01/2009
MN213159/BTV-3/ISR/ISR-2210/18/2018
KP821384/BTV-1/SDN/SUD1987/01/1987
KP821360/BTV-1/CMR/CAR1982/01/1982
KP821445/BTV-9/ITL2003/01/2003
MZ395196/BTV-9/ZAF/2766/1942
98
MT078273/BTV-8/ZAF/Utrecht 1955/1937
MG255556/BTV-1/ZAF/Elliot VR13/2017
AM778439/BTV-4/ZAF/RSAvvvv/04
KP821409/BTV-4/EGY1977/01/1977
99
MG255466/BTV-4/ZAF/13032016/2016
86
MT078253/BTV-6/ZAF/Strathene 1959/1959
GQ506476/BTV-6/NET2008/05/2008
81
JN848763/BTV-1/CHN/SZ97/1/1997
JX272383/BTV-1/ZAF/5012
KP821457/BTV-14/POL2012/01/2012
JQ972835/BTV-11/ZAF/REF/2010
JX272503/BTV-12/ZAF/75005
MT078293/BTV-10/PRT/Portugal 1956/1956
MT119857/BTV-1/ZAF/63/60/1960
96
MN710180/BTV-5/ZAF/RSArrrr/05/1953
JX272493/BTV-13/ZAF/160
MG255576/BTV-2/VR18 2017/2017
MN710175/BTV-5/ISR2009/14/2009
MT028383/BTV-2/ZAF/Bethal VR04/2017
97
MG255546/BTV-3/ZAF/Wesselsbron VR11/2017
MT028383/BTV-2/ZAF/VR04 2017/2017
MG255646/BTV-4/ZAF/VR39 2017/2017
MK348541/BTV-3/ITL/SAR2018/2018
78
77
OM502356/BTV-1/ISR/ISR-2050/19/2019
MH383093/BTV-6/ISR/ISR-2095/3/17/2017
KP821460/BTV-15/ISR2006/11/2006
KP821422/BTV-24/ISR2008/02/2008
MT043232/BTV-7/ZAF/VR43/2017
KP196607/BTV/ZAF/BT 57/08/2008
97
95
OK507979/BTV-9/ISR/ISR-1763/3/19/2019
ON087703/BTV/ISR-2237/18/2018
KY513438/BTV-2/ISR-1894/12/2012
86
MN710178/BTV-2/ISR/ISR2010/33/2010
JX272393/BTV-22/ZAF/84/184
75
MW001815/BTV-1/GUY/BTV-1/16.03(7341)/2016
KX164023/BTV-1/USA/FL 10-044273/2010
KP821448/BTV-9/LBY/LIB2008/08/2008
95
KP696576/BTV-1/IND/IND2003/10/2003
94
KP696626/BTV-9/IND/IND2004/04/2004
93
KF664127/BTV-1/IND/NRT37/ABT/HSR/2008
MN698765/BTV-1/AUS/V8776/2012
99
KP821453/BTV-9/TUR/TUR1998/02/1998
72
KP821441/BTV-9/BUL1999/01/1999
AM778438/BTV-1/GRC/GRE2001/07/2001
97
MK893205/BTV-16/ISR/ISR-2101/15/2015
70
KP821347/BTV-16/CYP/CYP2004/01/2004
MH559812/BTV-28/1537/14/2014
0.050

(d)

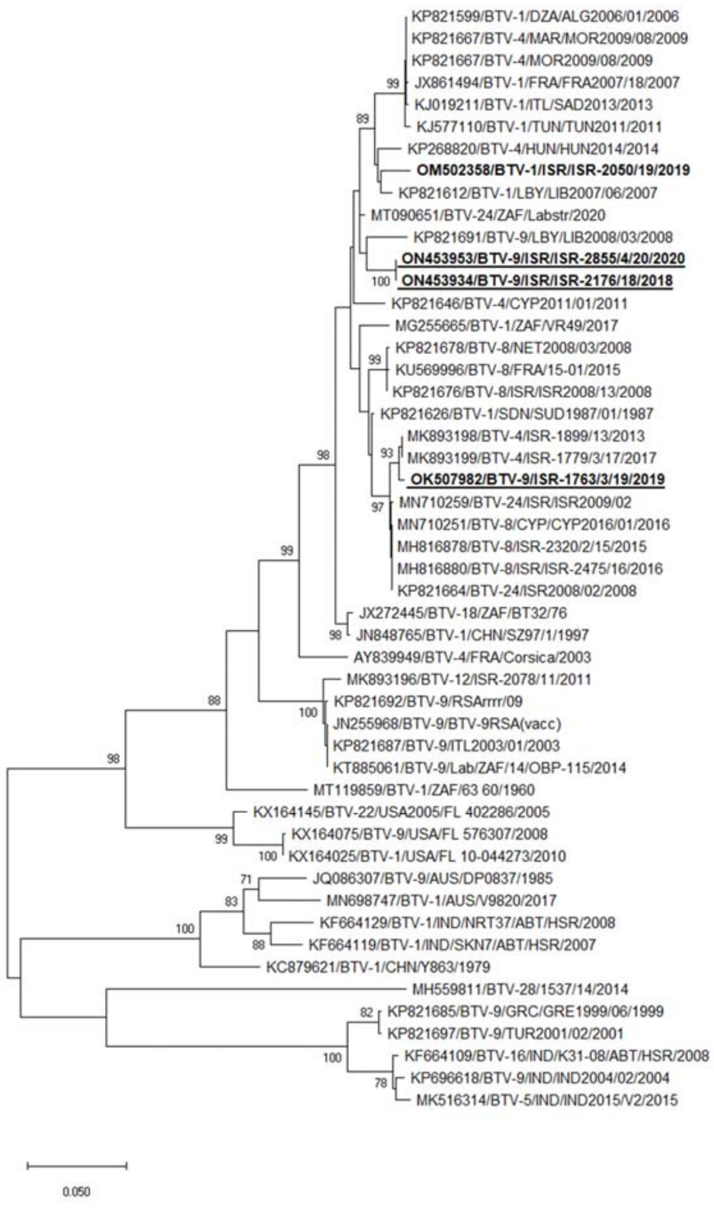
KP821599/BTV-1/DZA/ALG2006/01/2006
KP821667/BTV-4/MAR/MOR2009/08/2009
KP821667/BTV-4/MOR2009/08/2009
JX861494/BTV-1/FRA/FRA2007/18/2007
KJ019211/BTV-1/ITL/SAD2013/2013
KJ577110/BTV-1/TUN/TUN2011/2011
KP268820/BTV-4/HUN/HUN2014/2014
OM502358/BTV-1/ISR/ISR-2050/19/2019
KP821612/BTV-1/LBY/LIB2007/06/2007
MT090651/BTV-24/ZAF/Labstr/2020
KP821691/BTV-9/LBY/LIB2008/03/2008
ON453953/BTV-9/ISR/ISR-2855/4/20/2020
ON453934/BTV-9/ISR/ISR-2176/18/2018
KP821646/BTV-4/CYP2011/01/2011
MG255665/BTV-1/ZAF/VR49/2017
KP821678/BTV-8/NET2008/03/2008
KU569996/BTV-8/FRA/15-01/2015
KP821676/BTV-8/ISR/ISR2008/13/2008
KP821626/BTV-1/SDN/SUD1987/01/1987
MK893198/BTV-4/ISR-1899/13/2013
MK893199/BTV-4/ISR-1779/3/17/2017
OK507982/BTV-9/ISR-1763/3/19/2019
MN710259/BTV-24/ISR/ISR2009/02
MN710251/BTV-8/CYP/CYP2016/01/2016
MH816878/BTV-8/ISR-2320/2/15/2015
MH816880/BTV-8/ISR/ISR-2475/16/2016
KP821664/BTV-24/ISR2008/02/2008
JX272445/BTV-18/ZAF/BT32/76
JN848765/BTV-1/CHN/SZ97/1/1997
AY839949/BTV-4/FRA/Corsica/2003
MK893196/BTV-12/ISR-2078/11/2011
KP821692/BTV-9/RSArrrr/09
JN255968/BTV-9/BTV-9RSA(vacc)
KP821687/BTV-9/ITL2003/01/2003
KT885061/BTV-9/Lab/ZAF/14/OBP-115/2014
MT119859/BTV-1/ZAF/63 60/1960
KX164145/BTV-22/USA2005/FL 402286/2005
KX164075/BTV-9/USA/FL 576307/2008
KX164025/BTV-1/USA/FL 10-044273/2010
JQ086307/BTV-9/AUS/DP0837/1985
MN698747/BTV-1/AUS/V9820/2017
KF664129/BTV-1/IND/NRT37/ABT/HSR/2008
KF664119/BTV-1/IND/SKN7/ABT/HSR/2007
KC879621/BTV-1/CHN/Y863/1979
MH559811/BTV-28/1537/14/2014
KP821685/BTV-9/GRC/GRE1999/06/1999
KP821697/BTV-9/TUR2001/02/2001
KF664109/BTV-16/IND/K31-08/ABT/HSR/2008
KP696618/BTV-9/IND/IND2004/02/2004
MK516314/BTV-5/IND/IND2015/V2/2015
0.050

(e)

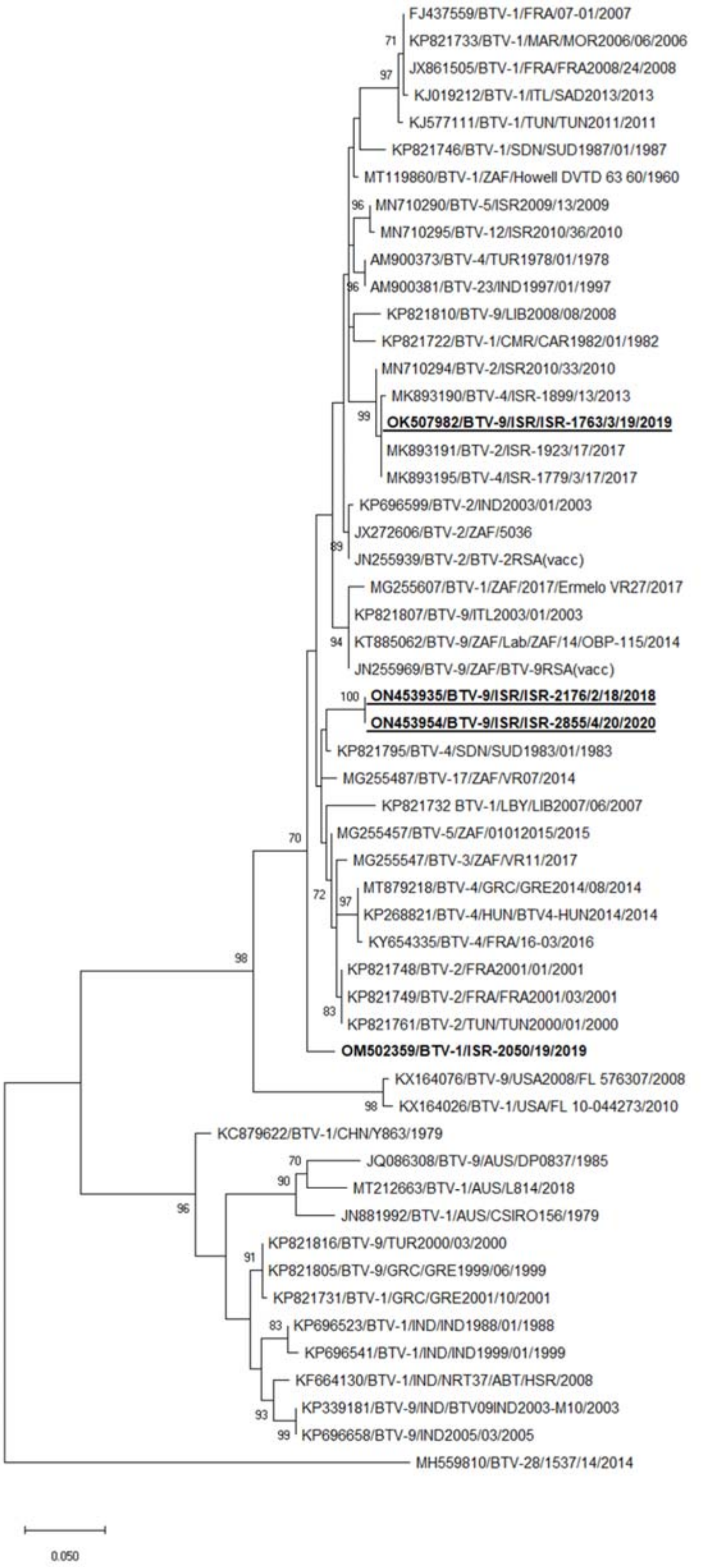

FJ437559/BTV-1/FRA/07-01/2007
KP821733/BTV-1/MAR/MOR2006/06/2006
JX861505/BTV-1/FRA/FRA2008/24/2008
KJ019212/BTV-1/ITL/SAD2013/2013
KJ577111/BTV-1/TUN/TUN2011/2011
KP821746/BTV-1/SDN/SUD1987/01/1987
MT119860/BTV-1/ZAF/Howell DVTD 63 60/1960
MN710290/BTV-5/ISR2009/13/2009
MN710295/BTV-12/ISR2010/36/2010
AM900373/BTV-4/TUR1978/01/1978
AM900381/BTV-23/IND1997/01/1997
KP821810/BTV-9/LIB2008/08/2008
KP821722/BTV-1/CMR/CAR1982/01/1982
MN710294/BTV-2/ISR2010/33/2010
MK893190/BTV-4/ISR-1899/13/2013
OK507982/BTV-9/ISR/ISR-1763/3/19/2019
MK893191/BTV-2/ISR-1923/17/2017
MK893195/BTV-4/ISR-1779/3/17/2017
KP696599/BTV-2/IND2003/01/2003
JX272606/BTV-2/ZAF/5036
JN255939/BTV-2/BTV-2RSA(vacc)
MG255607/BTV-1/ZAF/2017/Ermelo VR27/2017
KP821807/BTV-9/ITL2003/01/2003
KT885062/BTV-9/ZAF/Lab/ZAF/14/OBP-115/2014
JN255969/BTV-9/ZAF/BTV-9RSA(vacc)
ON453935/BTV-9/ISR/ISR-2176/2/18/2018
ON453954/BTV-9/ISR/ISR-2855/4/20/2020
KP821795/BTV-4/SDN/SUD1983/01/1983
MG255487/BTV-17/ZAF/VR07/2014
KP821732 BTV-1/LBY/LIB2007/06/2007
MG255457/BTV-5/ZAF/01012015/2015
MG255547/BTV-3/ZAF/VR11/2017
MT879218/BTV-4/GRC/GRE2014/08/2014
KP268821/BTV-4/HUN/BTV4-HUN2014/2014
KY654335/BTV-4/FRA/16-03/2016
KP821748/BTV-2/FRA2001/01/2001
KP821749/BTV-2/FRA/FRA2001/03/2001
KP821761/BTV-2/TUN/TUN2000/01/2000
OM502359/BTV-1/ISR-2050/19/2019
KX164076/BTV-9/USA2008/FL 576307/2008
KX164026/BTV-1/USA/FL 10-044273/2010
KC879622/BTV-1/CHN/Y863/1979
JQ086308/BTV-9/AUS/DP0837/1985
MT212663/BTV-1/AUS/L814/2018
JN881992/BTV-1/AUS/CSIRO156/1979
KP821816/BTV-9/TUR2000/03/2000
KP821805/BTV-9/GRC/GRE1999/06/1999
KP821731/BTV-1/GRC/GRE2001/10/2001
KP696523/BTV-1/IND/IND1988/01/1988
KP696541/BTV-1/IND/IND1999/01/1999
KF664130/BTV-1/IND/NRT37/ABT/HSR/2008
KP339181/BTV-9/IND/BTV09IND2003-M10/2003
KP696658/BTV-9/IND2005/03/2005
MH559810/BTV-28/1537/14/2014
0.050

(f)

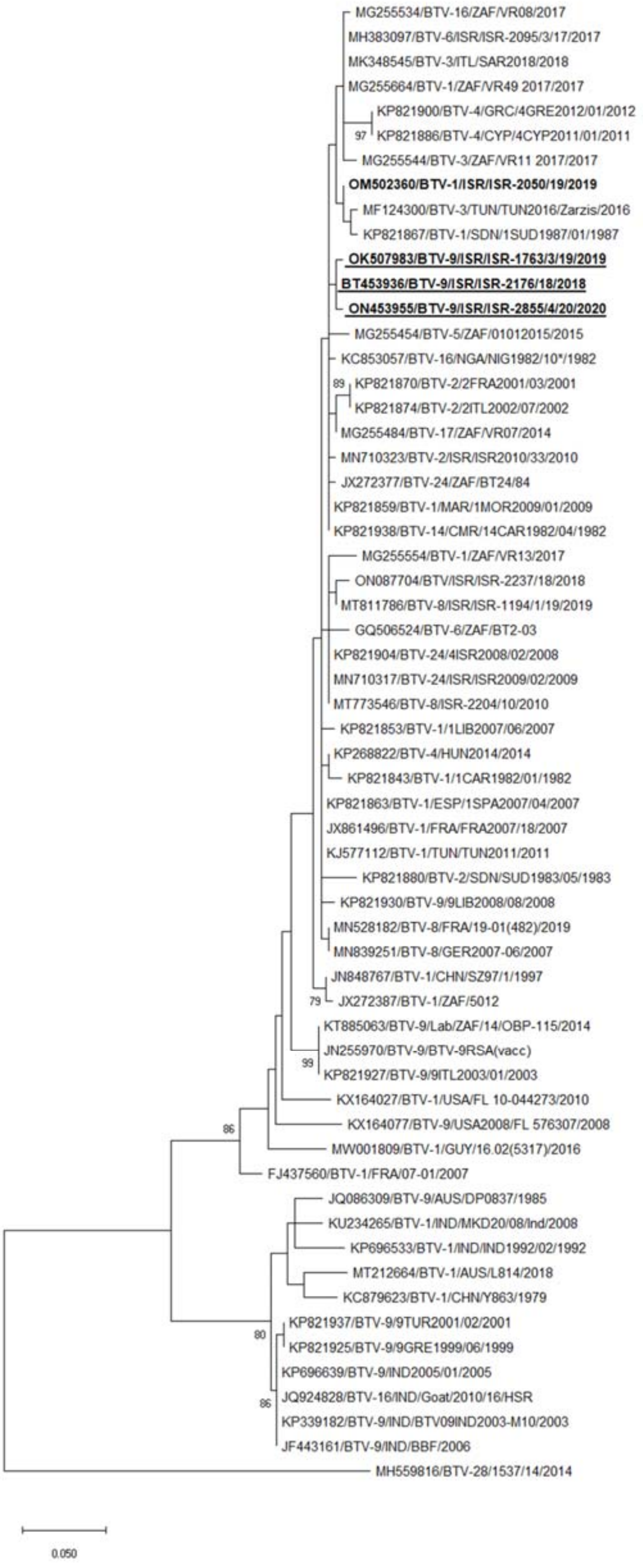
MG255534/BTV-16/ZAF/VR08/2017
MH383097/BTV-6/ISR/ISR-2095/3/17/2017
MK348545/BTV-3/ITL/SAR2018/2018
MG255664/BTV-1/ZAF/VR49 2017/2017
KP821900/BTV-4/GRC/4GRE2012/01/2012
97
KP821886/BTV-4/CYP/4CYP2011/01/2011
MG255544/BTV-3/ZAF/VR11 2017/2017
OM502360/BTV-1/ISR/ISR-2050/19/2019
MF124300/BTV-3/TUN/TUN2016/Zarzis/2016
KP821867/BTV-1/SDN/1SUD1987/01/1987
OK507983/BTV-9/ISR/ISR-1763/3/19/2019
BT453936/BTV-9/ISR/ISR-2176/18/2018
ON453955/BTV-9/ISR/ISR-2855/4/20/2020
MG255454/BTV-5/ZAF/01012015/2015
KC853057/BTV-16/NGA/NIG1982/10*/1982
89
KP821870/BTV-2/2FRA2001/03/2001
KP821874/BTV-2/2ITL2002/07/2002
MG255484/BTV-17/ZAF/VR07/2014
MN710323/BTV-2/ISR/ISR2010/33/2010
JX272377/BTV-24/ZAF/BT24/84
KP821859/BTV-1/MAR/1MOR2009/01/2009
KP821938/BTV-14/CMR/14CAR1982/04/1982
MG255554/BTV-1/ZAF/VR13/2017
ON087704/BTV/ISR/ISR-2237/18/2018
MT811786/BTV-8/ISR/ISR-1194/1/19/2019
GQ506524/BTV-6/ZAF/BT2-03
KP821904/BTV-24/4ISR2008/02/2008
MN710317/BTV-24/ISR/ISR2009/02/2009
MT773546/BTV-8/ISR-2204/10/2010
KP821853/BTV-1/1LIB2007/06/2007
KP268822/BTV-4/HUN2014/2014
KP821843/BTV-1/1CAR1982/01/1982
KP821863/BTV-1/ESP/1SPA2007/04/2007
JX861496/BTV-1/FRA/FRA2007/18/2007
KJ577112/BTV-1/TUN/TUN2011/2011
KP821880/BTV-2/SDN/SUD1983/05/1983
KP821930/BTV-9/9LIB2008/08/2008
MN528182/BTV-8/FRA/19-01(482)/2019
MN839251/BTV-8/GER2007-06/2007
JN848767/BTV-1/CHN/SZ97/1/1997
79
JX272387/BTV-1/ZAF/5012
KT885063/BTV-9/Lab/ZAF/14/OBP-115/2014
JN255970/BTV-9/BTV-9RSA(vacc)
99
KP821927/BTV-9/9ITL2003/01/2003
KX164027/BTV-1/USA/FL 10-044273/2010
KX164077/BTV-9/USA2008/FL 576307/2008
86
MW001809/BTV-1/GUY/16.02(5317)/2016
FJ437560/BTV-1/FRA/07-01/2007
JQ086309/BTV-9/AUS/DP0837/1985
KU234265/BTV-1/IND/MKD20/08/Ind/2008
KP696533/BTV-1/IND/IND1992/02/1992
MT212664/BTV-1/AUS/L814/2018
KC879623/BTV-1/CHN/Y863/1979
KP821937/BTV-9/9TUR2001/02/2001
80
KP821925/BTV-9/9GRE1999/06/1999
KP696639/BTV-9/IND2005/01/2005
JQ924828/BTV-16/IND/Goat/2010/16/HSR
86
KP339182/BTV-9/IND/BTV09IND2003-M10/2003
JF443161/BTV-9/IND/BBF/2006
MH559816/BTV-28/1537/14/2014
0.050

(g)

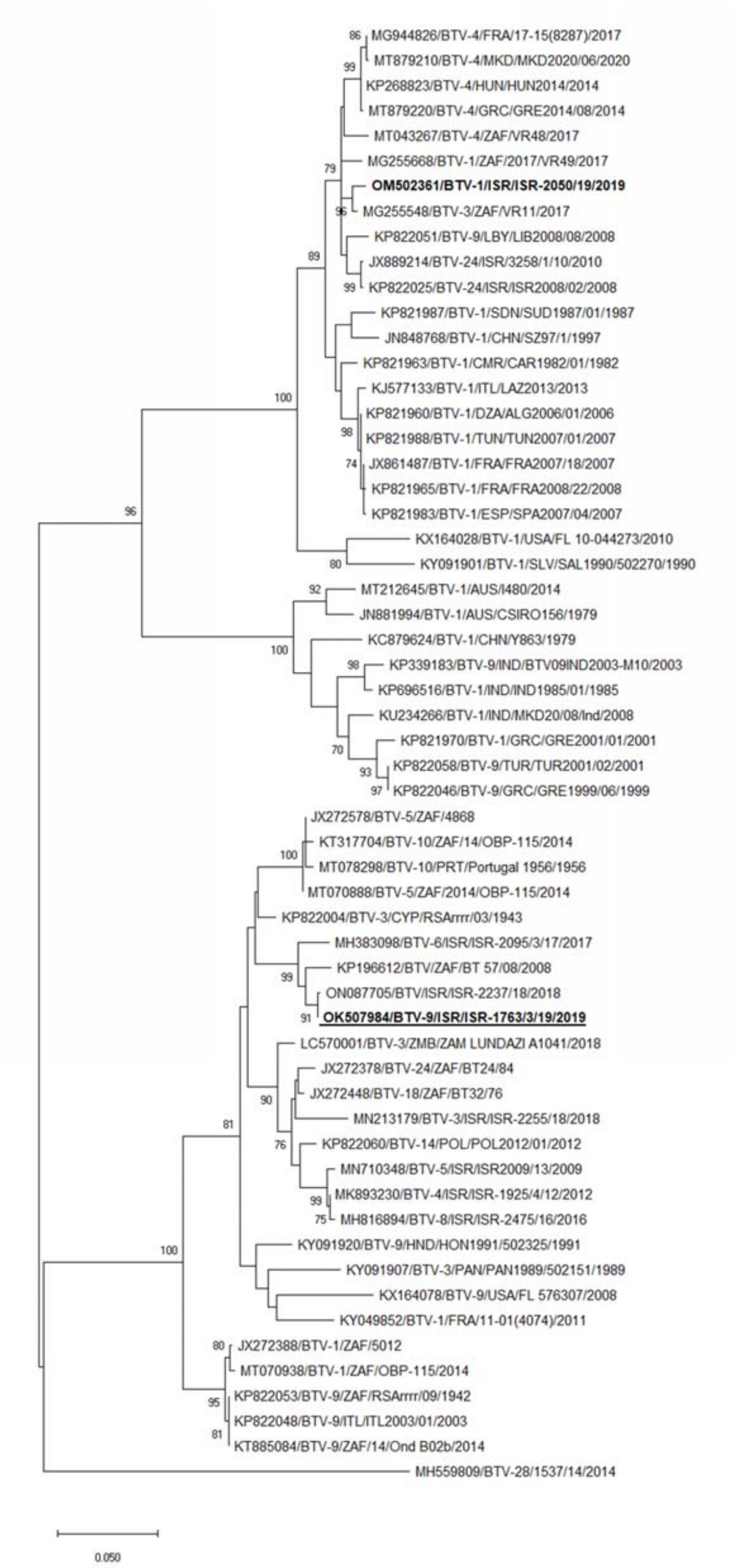
MG944826/BTV-4/FRA/17-15(8287)/2017
MT879210/BTV-4/MKD/MKD2020/06/2020
KP268823/BTV-4/HUN/HUN2014/2014
MT879220/BTV-4/GRC/GRE2014/08/2014
MT043267/BTV-4/ZAF/VR48/2017
MG255668/BTV-1/ZAF/2017/VR49/2017
OM502361/BTV-1/ISR/ISR-2050/19/2019
MG255548/BTV-3/ZAF/VR11/2017
KP822051/BTV-9/LBY/LIB2008/08/2008
JX889214/BTV-24/ISR/3258/1/10/2010
KP822025/BTV-24/ISR/ISR2008/02/2008
KP821987/BTV-1/SDN/SUD1987/01/1987
JN848768/BTV-1/CHN/SZ97/1/1997
KP821963/BTV-1/CMR/CAR1982/01/1982
KJ577133/BTV-1/ITL/LAZ2013/2013
KP821960/BTV-1/DZA/ALG2006/01/2006
KP821988/BTV-1/TUN/TUN2007/01/2007
JX861487/BTV-1/FRA/FRA2007/18/2007
KP821965/BTV-1/FRA/FRA2008/22/2008
KP821983/BTV-1/ESP/SPA2007/04/2007
KX164028/BTV-1/USA/FL 10-044273/2010
KY091901/BTV-1/SLV/SAL1990/502270/1990
MT212645/BTV-1/AUS/I480/2014
JN881994/BTV-1/AUS/CSIRO156/1979
KC879624/BTV-1/CHN/Y863/1979
KP339183/BTV-9/IND/BTV09IND2003-M10/2003
KP696516/BTV-1/IND/IND1985/01/1985
KU234266/BTV-1/IND/MKD20/08/Ind/2008
KP821970/BTV-1/GRC/GRE2001/01/2001
KP822058/BTV-9/TUR/TUR2001/02/2001
KP822046/BTV-9/GRC/GRE1999/06/1999
JX272578/BTV-5/ZAF/4868
KT317704/BTV-10/ZAF/14/OBP-115/2014
MT078298/BTV-10/PRT/Portugal 1956/1956
MT070888/BTV-5/ZAF/2014/OBP-115/2014
KP822004/BTV-3/CYP/RSArrrr/03/1943
MH383098/BTV-6/ISR/ISR-2095/3/17/2017
KP196612/BTV/ZAF/BT 57/08/2008
ON087705/BTV/ISR/ISR-2237/18/2018
OK507984/BTV-9/ISR/ISR-1763/3/19/2019
LC570001/BTV-3/ZMB/ZAM LUNDAZI A1041/2018
JX272378/BTV-24/ZAF/BT24/84
JX272448/BTV-18/ZAF/BT32/76
MN213179/BTV-3/ISR/ISR-2255/18/2018
KP822060/BTV-14/POL/POL2012/01/2012
MN710348/BTV-5/ISR/ISR2009/13/2009
MK893230/BTV-4/ISR/ISR-1925/4/12/2012
MH816894/BTV-8/ISR/ISR-2475/16/2016
KY091920/BTV-9/HND/HON1991/502325/1991
KY091907/BTV-3/PAN/PAN1989/502151/1989
KX164078/BTV-9/USA/FL 576307/2008
KY049852/BTV-1/FRA/11-01(4074)/2011
JX272388/BTV-1/ZAF/5012
MT070938/BTV-1/ZAF/OBP-115/2014
KP822053/BTV-9/ZAF/RSArrrr/09/1942
KP822048/BTV-9/ITL/ITL2003/01/2003
KT885084/BTV-9/ZAF/14/Ond B02b/2014
MH559809/BTV-28/1537/14/2014
0.050

(h)

Figure S1. Phylogenetic trees of Israeli BTV-1 and BTV-9 strains isolated in 2018-2020 and global strains. a) segment 1; b) segment 3; c) segment 4; d) segment 5; e) segment 7; f) segment 8; g) segment 9; e) segment 10. Israeli BTV-1 strain is shown in bold. Israeli BTV-9 strain is shown in bold and is underlined. The phylogeny was inferred using the Maximum Likelihood method and Tamura-Nei model method. The percentage of replicate trees in which the associated taxa clustered together in the bootstrap test (1000 replicates) are shown next to the branches. Viruses were identified by accession number/serotype/location/isolate/year.

Table S1. List of primers used for partial sequencing of Israeli bluetongue virus serotype 9

segment	name	sequence	length of product	source
	UNI-VP1-1-F	GTTAAAATGCAATGGTCGCAAT		this study
1	VP1-414R	TAT ATT TGC ATC TCG TTT TTA GC	436	this study
	UNI-VP1-810-R	CCCCACATCTTYACAAACCA	829	this study
	VP1-Uni-2212F	ATA ACA TGG CTA TTG GGA CC		this study
	VP1-Uni-2684R	TCA AAC TTT CTG TCG CGA TAC	493	this study
2	9VP2-1F	GTT AAA AAC GCT GTC CCG AGA		this study
	9VP2-422R	TTC GCA ACM CCA TTC ATA AA	444	this study
	9VP2-950R	TCC GAG AAG AAC ATG GTA TCA	970	this study
	9VP2-150F	AAA GAG CGT AAA GCA ATT GA		this study
	9VP2-507R	CAC ATC TTG TAC CCT GGA ATG	377	this study
3	VP3-Isr-F	GGAGATGTRCTKTCGAGTGATTC		this study
	VP3-Isr-R	CATTAAGYTGCTGTGCGGTTGG	958	this study
	BT-S3-F	GTTAAATTTCCGTRGCYATGGC		Sun 2014
	VP3-337R	CCT GCG ACA TCT CTT CAT AGT	352	this study
4	BT-S4-F	GTTAAAACATGCCTGAGCCA		this study
	VP4-281R	ATC TTG ACT TTT TGC GAC TTC C	303	this study
	VP4-516R	TCA GTH GGC TCA TCG TCA AA	536	this study
5	NS1-1191F	TAG TTC GCG ATG AAC AAA TTG G		this study
	uni-NS1-1747R	AAG TTG AAA AGT TCT AGT AGA GTG CT	579	this study
	UNI-NS1-40F	GCGCTTTTTGAGAAAATACAAC		this study
	UNI-NS1-428R	AGAGAATCATCCAATCTAACTCT	411	this study
6	9VP5-1F	GTT AAA AAG CGT ACC CTT AGC		this study
	9VP5-818R	TCA AAT CAA TTC CGC TCA G	836	this study
	9VP5-442R	TTC GCA ACM CCA TTC ATA AA	431	this study
7	VP7-Isr-F	TTTATGTGTTTAGATATGATGCTGT	874	this study
	3VP7-1f-deg	GTTAAAAATHTCTAGAGATGGACACT	694	this study
	VP7-Isr-R	TATTCGGCAGTGTAGTTCTGTTTAG		this study

8	BT-S8-F	GTTAAAAAAWCCTTGAGTCATG		Sun 2014
	NS2-331R	TCA ACC ATC ACD CCA TTA TGT T	353	this study
	3NS2-350R	GCA TCA ACC ATC ACA CCA TT	370	this study
	UNI-NS2-17F	ATGGAGCAAAAGCAACG		this study
	UNI-NS2-1026R	AGAGACAAAAGCAACACGCT	1026	this study
9	BT-S9-F	GTTAAAAAATCGCATATGTCRG		Sun 2014
	VP6-448R	TCA ACT TTC GTA CCG TAT TTA GA	471	this study
10	BTV-NS3-183F	AAATMTTGGAYAAAGCRATGTCAAA		Wernike 2015
	NS3-802r-deg	TAA GTG TGT AGY GYC GCG YA	639	this study
	BT-EHD-S10R	ACCCTCCCCCGYTAKACARC	604	this study

Table S2. List of sequenced Israeli BTV strains used for the present study

host/date of sampling/source	segment/ length<br>serotype /strain	1/3944	2/2926-2939	3/2772	4/1981	5/1774-1776	6/1637-1645	7/1156	8/1125-1131	9/1047-1050	10/822
sheep/2018/<br>whole blood	9/ISR-2077/3/18	ON453918<br>12-436	ON453919<br>1-574	ON453920<br>18-357	ON453921<br>1-535	ON453922<br>52-435	ON453923<br>46-790	ON453924<br>14-526	ON453925<br>11-354	ON453926<br>1-470	ON453927<br>120-783
cattle/2018/<br>whole blood	9/ISR-2176/2/18	ON453928<br>12-436	ON453929<br>153-518	ON453930<br>18-357	ON453931<br>24-286	ON453932<br>1231-1712	ON453933<br>2-441	ON453934<br>214-888	ON453935<br>1-354	ON453936<br>22-465	ON453937<br>1-282
sheep/2019/<br>VI (Vero cells)	9/ISR-1763/3/19	OK507975<br>1-3930	OK507976<br>10-2893	OK507977<br>4-2768	OK507978<br>8-1968	OK507979<br>1-1754	OK507980<br>13-1625	OK507981<br>13-1114	OK507982<br>1-1112	OK507983<br>1-1036	OK507984<br>14-794
sheep/2019/<br>VI (ECE)	9/ISR-1872/19	-	-	-	-	-	-	ON453957<br>14-881	ON453958<br>14-1031	ON453959<br>3-1018	-
sheep/2019/<br>VI (ECE)	9/ISR-1915/19	-	-	-	-	-	-	ON453960<br>15-884	ON453961<br>11-1017	ON453963<br>5-1008	-
cattle/2019/<br>VI (ECE)	9/ISR-2020/2/19	ON453908<br>1-817	ON453909<br>153-949	ON453910<br>91-1015	ON453911<br>1-535	ON453912<br>49-435	ON453913<br>1-829	ON453914<br>1-874	ON453915<br>11-1017	ON453916<br>1-471	ON453917<br>200-802
sheep/2019/<br>VI (ECE)	9/ISR-2077/4/19	-	-	-	-	-	-	ON453963<br>16-885	ON453964<br>42-998	ON453965<br>49-1012	-
sheep/2020/<br>whole blood	9/ISR-2758/1/20	ON453938<br>1-436	ON453939<br>153-528	ON453940<br>2196-2758	ON453941<br>1-257	ON453942<br>1235-1713	ON494587<br>1-441	ON453943<br>246-884	ON453944<br>11-354	ON453945<br>1-470	ON453946<br>200-783
cattle/2020/	9/ISR-2855/4/20	ON453947	ON453948	ON453949	ON453950	ON453951	ON453952	ON453953	ON453954	ON453955	ON453956

whole blood		1-817	17-949	18-1015	1-535	52-435	1-441	203-884	11-354	1-470	200-783
cattle/2019	1/ISR-2050/19	OM502362	OM502353	OM502354	OM502355	OM502356	OM502357	OM502358	OM502359	OM502360	OM502361
VI (Vero cells)		15-3933	5-2768	5-2768	1-1969	1-1771	11-1631	1-1143	17-1113	1-1044	1-822

Upper rows provide accession numbers of sequenced regions. Lower rows show sequences regions. Source- source or sequencing. VI- virus isolation. ECE- embryonated chicken embryos. Strains ISR-2050/19 and ISR-1763/3/19 were sequenced by NGS technology; the last of the strains were sequenced by Sanger sequencing technology.
